# Biological Consequences of Ancient Gene Acquisition and Duplication in the Large Genome of *Candidatus* Solibacter usitatus Ellin6076

**DOI:** 10.1371/journal.pone.0024882

**Published:** 2011-09-15

**Authors:** Jean F. Challacombe, Stephanie A. Eichorst, Loren Hauser, Miriam Land, Gary Xie, Cheryl R. Kuske

**Affiliations:** 1 Bioscience Division, Los Alamos National Laboratory, Los Alamos, New Mexico, United States of America; 2 Genome Analysis and Systems Modeling Group, Biosciences Division, Oak Ridge National Laboratory, Oak Ridge, Tennessee, United States of America; Biodiversity Insitute of Ontario - University of Guelph, Canada

## Abstract

Members of the bacterial phylum *Acidobacteria* are widespread in soils and sediments worldwide, and are abundant in many soils. Acidobacteria are challenging to culture *in vitro,* and many basic features of their biology and functional roles in the soil have not been determined. *Candidatus* Solibacter usitatus strain Ellin6076 has a 9.9 Mb genome that is approximately 2–5 times as large as the other sequenced *Acidobacteria* genomes. Bacterial genome sizes typically range from 0.5 to 10 Mb and are influenced by gene duplication, horizontal gene transfer, gene loss and other evolutionary processes. Our comparative genome analyses indicate that the Ellin6076 large genome has arisen by horizontal gene transfer via ancient bacteriophage and/or plasmid-mediated transduction, and widespread small-scale gene duplications, resulting in an increased number of paralogs. Low amino acid sequence identities among functional group members, and lack of conserved gene order and orientation in regions containing similar groups of paralogs, suggest that most of the paralogs are not the result of recent duplication events. The genome sizes of additional cultured *Acidobacteria* strains were estimated using pulsed-field gel electrophoresis to determine the prevalence of the large genome trait within the phylum. Members of subdivision 3 had larger genomes than those of subdivision 1, but none were as large as the Ellin6076 genome. The large genome of Ellin6076 may not be typical of the phylum, and encodes traits that could provide a selective metabolic, defensive and regulatory advantage in the soil environment.

## Introduction

Soils contain an abundant and diverse array of bacteria that are critical for plant life and nutrient cycling in terrestrial ecosystems. *Acidobacteria*, one of the most widespread and abundant phyla found in soils and sediments worldwide [1], [2], [3], comprise up to 50% of the rRNA gene sequences from bacterial clone libraries in some soils [4]. They have also been found in a variety of other environments, including aquatic [5], [6], extreme [7], [8], and polluted environments [9], and wastewater systems [10], [11]. Their phylogenetic diversity, common occurrence and widespread abundance suggest that *Acidobacteria* may be important contributors in a variety of ecosystems.

The *Acidobacteria* phylum is defined by a large collection of 16S rRNA gene sequences (>8,000 in the ARB_SILVA Database (August 2011) [12]) that fall into 26 major subdivisions [9]. However members of this phylum have been difficult to isolate *in vitro*. Cultured isolates are slow growing and difficult to maintain, which has hampered their biological and physiological characterization [8], [13], [14], [15], [16], [17], [18], [19], [20], [21]. Despite their widespread occurrence in nature, much about *Acidobacteria* biology and potential ecological roles in soil remain unknown.

Comparative analysis of three sequenced *Acidobacteria* genomes from subdivisions 1 and 3 revealed that the genome of the subdivision 3 member, *Candidatus* Solibacter usitatus Ellin6076 (hereafter termed Ellin6076), is 9.9 Mb in size whereas the genomes of subdivision 1 strains, *Candidatus* Korebacter versatilis Ellin345 (hereafter termed Ellin345) and *Acidobacterium capsulatum* are about half the size (5.7 Mb and 4.1Mb, respectively) [22].

Variations in genome size, structure and gene arrangement impact bacterial phenotype and contribute to genome evolution [23]. Genome size can differ dramatically within the same genus or family, and is not associated with specific bacterial lineages or phenotypes. Large genomes (defined here as >7 Mb) are found in many diverse species across the Domain *Bacteria* [24], [25]. In contrast, obligatory host-associated bacterial pathogens, insect symbionts and extremophiles have lost genes during specialization for their environments, typically harboring reduced genomes compared to facultative pathogens and free-living bacteria [26], [27]. Free living soil and marine species tend to have larger genomes [25], [28], presumably providing a selective advantage in highly variable, changing environments [29].

The mechanisms that influence genome size, structure and evolution include horizontal gene transfer events, and large and small scale sequence duplications [30], [31]. Horizontal transfer is a mechanism for homolog acquisition, and gene duplication can often lead to paralogs, which are redundant copies of genes that can undergo mutations leading to functional diversification [30], [31], [32], [33]. We hypothesized that identifying the mechanisms that shaped the large genome of Ellin6076, and its distinctive physiological features, would provide information about its potential biological and ecological roles in the soil. In this study, the genomic features of the Ellin6076 genome were examined to identify potential past horizontal transfer events, and to catalogue the metabolic and regulatory traits encoded in paralogs.

## Results

### Genomic features

#### Repeat sequences

Repeat sequence analysis indicated that numerous short repeat sequences in the Ellin6076 genome contribute to its large size. The total number of repeats identified in Ellin6076 was 8.7-fold greater than in Ellin345 (Table S1). However, most of the repeats identified in both genomes were less than 50–100 nucleotides long. Consistent with this finding, comparison of the Ellin6076 genome sequence against itself using nucmer [34] (Figure S1) shows only the identity line, demonstrating that long repeat regions and/or whole genome duplication are not present in the large genome of Ellin6076. If there had been long repeat regions, they would have shown up in the plot as shorter parallel lines next to the center identity line.

#### GC content

**Figure 1:**
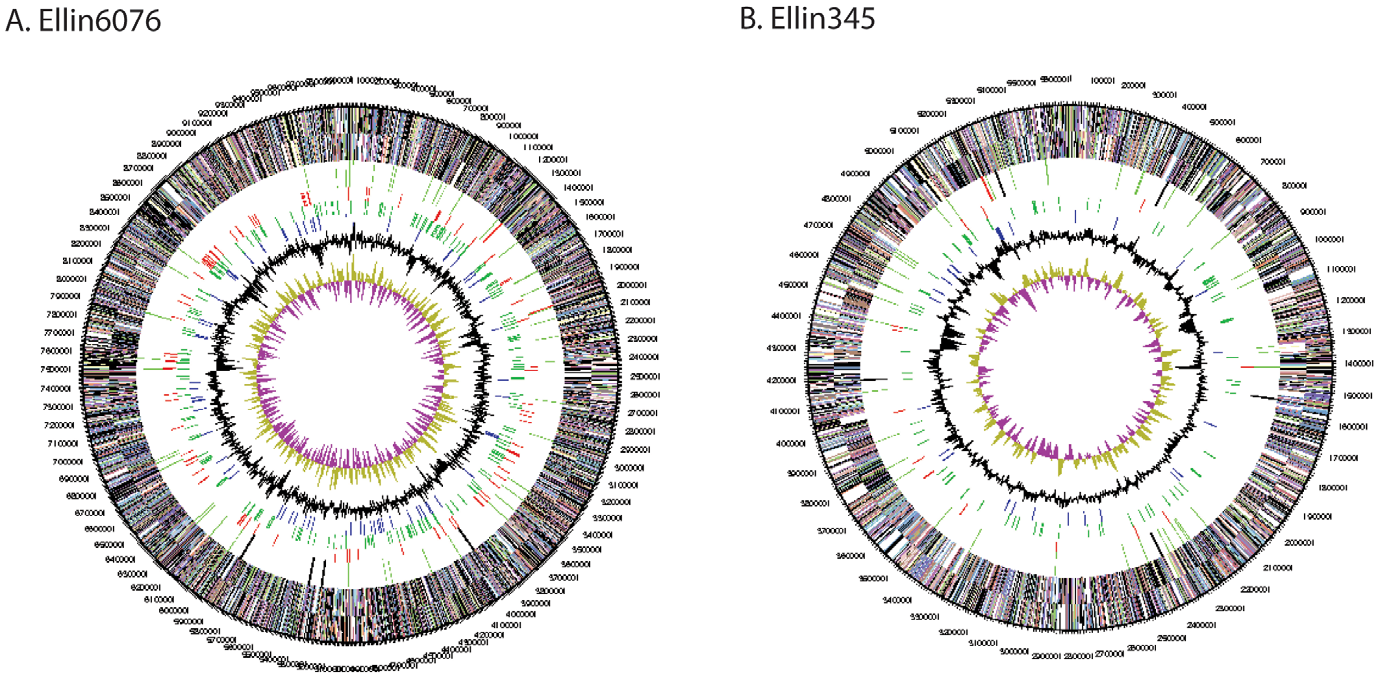
Circular map of the Ellin6076 (Panel A) and Ellin345 (Panel B) genomes obtained from the IMG system (http://img.doe.gov). From outside to the center: Circles 1 and 2: forward and reverse strand genes colored by COG categories; Circles 3 and 4: RNA genes (tRNAs green, sRNAs red, other RNAs black); Circles 5 and 6: mobile elements; Circle 7: GC content; Circle 8: GC skew. Colors representing the COG category codes and function definitions: cyan, [A] RNA processing and modification; light lime, [B] Chromatin structure and dynamics; light aqua, [C] Energy production and conversion; pale lavender, [D] Cell cycle control, cell division, chromosome partitioning; light crimson, [E] Amino acid transport and metabolism; light blue green, [F] Nucleotide transport and metabolism; dark pink, [G] Carbohydrate transport and metabolism; teal, [H] Coenzyme transport and metabolism; violet blue, [I] Lipid transport and metabolism; violet, [J] Translation, ribosomal structure and biogenesis; light olive, [K] Transcription; yellow, [L] Replication, recombination and repair; light brown, [M] Cell wall/membrane/envelope biogenesis; light pink, [N] Cell motility; light green, [O] Posttranslational modification, protein turnover, chaperones; orange, [P] Inorganic ion transport and metabolism; lime, [Q] Secondary metabolites biosynthesis, transport and catabolism; purple, [R] General function prediction only; aqua, [S] Function unknown; brown, [T] Signal transduction mechanisms; light blue, [U] Intracellular trafficking, secretion and vesicular transport; baby blue, [V] Defense mechanisms; lavender, [W] Extracellular structures; light red, [Y] Nuclear structure; lime green, [Z] Cytoskeleton.

The circular maps of the Ellin6076 and Ellin345 genomes depicted in Figure 1 illustrate their overall organization, including forward and reverse coding sequences, RNA genes, mobile elements, GC content, and GC skew. The cumulative GC skew (Figure S2), and output from IslandPath [35] (data not shown), support our finding that the Ellin6076 genome does not contain any large regions with GC content that differ significantly from the average content of the genome.

#### Mobile genetic elements

The Ellin6076 genome contained 123 mobile genetic element genes encoding phage integrases, transposases and IS elements, compared to 29 mobile element genes in Ellin345 (Figure 1). When compared via BLAST, numerous integrase, transposase and IS element protein sequences had high (97–100%) amino acid sequence identities, and we classified them into identity groups (Table S2). Given the high sequence similarity among the genes comprising each mobile element group, it appears that these genes may have recently duplicated and dispersed throughout the genome. Although we identified numerous phage integrase genes in the Ellin6076 genome, no intact prophage regions were found [22]. Although the presence of phage integrase genes indicates that bacteriophage-mediated transduction contributed to the genome of Ellin6076, the absence of intact prophage regions suggests that phage integration events did not occur recently. To further investigate whether past phage infection played a significant role in shaping the Ellin6076 genome, we looked for degenerate prophage regions using the criteria reported in [36]. We also tried to locate clustered, regularly interspaced, short palindromic repeats (CRISPRs), which can indicate past phage integrations [37], [38]. We did not find any degenerate prophages or CRISPRs in the genome, but we did identify three CRISPR-associated (Cas) protein genes (Acid_0892, Acid_0893 and Acid_0895), which are normally located adjacent to CRISPR regions [39]. Since CRISPRs do not persist in the host genome over time [40], these observations suggest that CRISPR acquisition event(s) occurred in the past, and the Cas genes were left in the genome. This adds further support to our conjecture that any phage transduction events that brought genes into the Ellin6076 genome were not recent.

#### Genomic islands

A genomic island (GI) is a previously motile region of a genome, which has become fixed. GIs are frequently inserted near tRNA genes and flanking repeat sequences, and often contain genes encoding mobile elements [41] and may also include niche-specific functions, such as virulence or metabolic traits. Since the Ellin6076 genome contained many mobile element genes, we examined these regions for candidate GIs using the output from IslandPath [35].

We identified sixteen candidate GIs encompassing small groups of genes with aberrant GC content, along with numerous tRNA genes and mobile genetic elements. The most notable candidate GI regions (III, XI, XIII and XV) (Table S3) had a below-average GC content, dinucleotide bias that covered nearly the entire region, tRNA genes, and mobile elements. In addition to abundant phage integrase genes, some putative GIs contained fragments of genes annotated as either phage or plasmid-related. All of the putative GIs included multiple genes annotated as hypothetical, with no similarity to any known sequences, suggesting that degeneration of the regions had already occurred, similar to the degeneration seen in prophage regions over time following insertion into a host genome [36].

#### Gene duplication and paralogs

There was striking evidence of gene duplication in the Ellin6076 genome, corresponding to an increased number of paralogs. After correcting for genome size, Ellin6076 contained a higher percentage of paralogs (67.8%, 5426 paralogs in 1103 paralog groups) than Ellin345 (52.6%, 2543 paralogs in 677 paralog groups, Table 1). The number of paralogs in many categories was 4-fold or greater (Tables 2 and S5), and included the mobile elements described above, along with genes involved in cell wall/membrane biogenesis, signal transduction, intracellular trafficking and secretion, defense mechanisms and metabolism. Paralogous sequences with the same functional definition (e.g. serine/theronine protein kinases) in Ellin6076, generally showed less than 50% amino acid identity in full-length alignments (data not shown). The COG functional categories of the paralogs in the large Ellin6076 genome compared to the more average sized Ellin345 and other representative larger-smaller genome pairs are shown in Tables S4, S5. The sizes of the larger genomes ranged from 6.99 to 9.97 Mb, while the smaller genome sizes were in the range 3.27 to 5.90 (Table S4). For all but one pair (*Ralstonia eutrophia* H16 vs. *Ralstonia solanacearum* UW551), the smaller genome was approximately half the size of the larger one. The functional categories described above may represent increased metabolic and regulatory redundancy and diversity encoded in the Ellin6076 genome compared to Ellin345 (Table 2). Increased numbers of paralogs with annotated functions related to metabolic breadth, cellular defense, and gene regulation were common. The full list is presented in Table S5.

**Table 1:** Paralogs in larger-smaller genome pairs#.

Classification	Large genome	Size (Mb)	# genes	# paralogs (% of genes)	Small genome	Size (Mb)	# genes	# paralogs (% of genes)
Acidobacteria	Ellin6076	9.97	8002	5426 (67.8%)	Ellin345	5.65	4834	2543 (52.6%)
Alpha proteobacteria	*Magnetospirillum magnetotacticum* MS-1*	9.21	10334	6113 (59.2%)	*Magnetospirillum magneticum* AMB-1	4.97	4667	2428 (52.0%)
Alpha proteobacteria	*Mesorhizobium loti* MAFF303099	7.6	7352	4396 (59.8%)	*Mesorhizobium* sp. BNC1	4.94	4686	2606 (55.6%)
Beta proteobacteria	*Ralstonia eutropha* H16	7.42	6702	4395 (65.6%)	*Ralstonia solanacearum* UW551*	5.9	4418	2267 (51.3%)
Gamma proteobacteria	*Hahella chejuensis* KCTC 2396	7.22	6862	3242 (47.2%)	*Oceanospirillum* sp. MED92*	3.87	3735	1721 (46.1%)
Actinobacteria	*Mycobacterium smegmatis* MC2 155	6.99	6925	4535 (65.5%)	*Mycobacterium leprae* TN	3.27	2752	490 (17.8%)

# Data from the Integrated Microbial Genomes (IMG) System; http://img.jgi.doe.gov.

*Draft genome data.

**Table 2:** Distribution of genes in COG categories for *Acidobacteria* strains Ellin6076 and Ellin345.

COG CATEGORY	Strain Ellin 6076	Strain Ellin 345	Fold Increase+
**Information storage and processing**			
(L) Replication, recombination, repair			
Site-specific recombinase XerD	32	6	5.3
Transposase and inactivated derivatives	64	10	6.4
**Cellular processes**			
(M) Cell wall/membrane biogenesis			
4-amino-4-deoxy-L-arabinose transferase and related	26	7	3.7
L-alanine-DL-glutamate epimerase and related	25	4	6.3
Periplasmic protease	13	2	6.5
Endopolygalacturonase	11	0	>11.0
Dihydropicolinate synthase/N-acetylneuraminate lyase	9	2	4.5
ABC-type polysaccharide/polyol phosphate export system, permease	6	0	>6.0
ABC-type polysaccharide/polyol phosphate export system, ATPase	6	0	>6.0
Membrane proteins related to metalloendopeptidases	5	1	5.0
Sortase	6	1	6.0
(T) Signal transduction mechanisms			
Antirepressor regulating drug resistance, signal transduction comp.	12	3	4.0
Bacteriophytochrome	9	2	4.5
(U) Intracellular trafficking, secretion			
Flp pilus assembly protein, ATPase CpaE	4	0	>4.0
Flp pilus assembly protein, ATPase CpaF	4	0	>4.0
(C) Energy production, conversion			
FAD/FMN-containing dehydrogenases	8	2	4.0
Carbon dioxide conc. mechanism/carboxysome shell proteins	9	0	>9.0
FOG:HEAT repeat	6	0	>6.0
Rieske Fe-S protein	5	0	>5.0
Predicted acetamidase/formamidase	4	0	>4.0
Cytochrome b subunit	4	0	>4.0
(E) Amino acid transport, metabolism			
Lysophospholipase L1 and related esterases	16	1	16.0
Dihydropicolinate synthase/N-acetylneuraminate lyase	9	2	4.5
Choliine dehydrogenase and related	8	2	4.0
Spermidine synthase	5	0	>5.0
Asparagine synthase (glutamate hydrolyzing)	4	0	>4.0
(G) Carbohydrate transport, metabolism			
Sugar phosphate isomerases/epimerases	41	6	6.8
Glucose dehydrogenase	26	0	>26.0
Gluconolactonase	13	2	6.5
Alpha-L-arabinofuranosidase	8	2	4.0
Alpha-L-fucosidase	7	1	7.0
Glucose/sorbosone dehydrogenases	6	1	6.0
ABC-type polysaccharide/polyol phosphate export system, permease	6	0	>6.0
ABC-type polysaccharide/polyol phosphate export system, ATPase	6	0	>6.0
2,4-dihydroxyhept-2-ene-1,7-dioic acid aldolase	5	0	>5.0
Beta-xylosidase	4	0	>4.0
Beta-galactosidase	4	1	4.0
(H) Coenzyme transport, metabolism			
2-polyprenyl-3-methyl-5-hydroxy-6-metoxy-1,4-benzoquinol methylase	18	4	4.5
Demethylmenaquinone methyltransferase	7	0	>7.0
(I) Lipid transport, metabolism			
Carboxylesterase type B	9	0	>9.0
(P) Inorganic ion transport, metabolism			
Arylsulfatase A and related enzymes	18	0	>18.0
Enterochelin esterase and related enzymes	16	2	8.0
Cytochrome c peroxidase	6	0	>6.0
(Q) Secondary metabolites			
Dienelactone hydrolase and related enzymes	8	1	8.0
Carbon dioxide conc. mechanism/carboxysome shell proteins	9	0	>9.0
Protein involved in biosynthesis of mitomycin antibiotics/fumonisin	4	0	>4.0
Predicted enzyme involved in methoxymalonyl-ACP biosynthesis	4	1	4.0

+ Not normalized for genome size.

Only categories with four-fold or greater differences are shown.

### Phylogenetic and dN/dS analysis of paralogs

Phylogenetic analysis was performed on 27 representative groups of paralogs that encompassed a variety of functions, including drug resistance, metabolism, protein binding, regulation and transport. In keeping with the low amino acid sequence identities revealed through BLAST analysis, nearly all of the paralogs within a particular group showed divergent evolutionary relationships in the phylogenetic tree (some examples are shown in Figures S3, S4). The mobile element paralogs were much more closely related in terms of sequence identities, and in two cases all of the sequences were identical. These results led to the question: Why would the Ellin6076 genome maintain so many paralogs with divergent sequences? To answer this, we performed a codon-based Z-test of positive selection (Table 3). The evolutionary pressures on protein coding sequences can be quantified by determining the ratio of substitution rates at synonymous and non-synonymous nucleotide sites [42]. The analysis was conducted for each pair of sequences, and as an overall average for all pairwise comparisons (Table 3). Three paralog groups, phage tail collar domain protein, CnaB-type protein and one phage integrase family protein group, had an average value of the test statistic Z above one, indicating positive values for selection among some of the sequences. Two paralog groups, the serine/threonine protein kinases and the two-component transcriptional regulator, winged helix family members, had no sequences showing positive selection. Some of the paralogs in the carboxylesterase type B group, and those with functions involved in transport, drug resistance and receptors, showed positive selection when compared to each other. Data for the pairwise comparisons of individual sequences in representative paralog groups are shown in Figures S5, S6, S7. Results of the substitution saturation tests performed on each paralog group are shown in Table 4. These results indicate that, while four paralog groups showed little substitution saturation (serine/threonine protein kinase, carboxylesterase, acetolactate synthase and one phage integrase family protein group), the sequences in the rest of the paralog groups did show substitution saturation and are therefore too divergent to be useful for further phylogenetic analyses.

**Table 3:** Results of codon-based test of positive selection, averaging over all sequence pairs.

Functional definition	Identifier of first sequence	dN-dS Stat from test of dN>dS (positive selection)	Probability
serine/threonine protein kinase	YP_821325	−6.478	1.000
two-component transcriptional regulator, winged helix family	YP_821372	−6.116	1.000
ABC transporter-related	YP_821380	0.218*	0.414
Carboxylesterase, type B	YP_821393	0.215*	0.415
Transcriptional repressor, CopY family	YP_821398	0.225*	0.411
Drug resistance transporter, EmrB/QacA subfamily	YP_821403	0.217*	0.414
TonB-dependent receptor, plug 1	YP_821405	−1.960*	1.000
TonB-dependent receptor, plug 2	YP_821493	−0.614*	1.000
anti-sigma factor antagonist	YP_821407	−1.275*	1.000
RNA polymerase, sigma-24 subunit, ECF subfamily	YP_821437	−0.670	1.000
phage tail collar domain protein	YP_821449	3.703*	0.000
oxidoreductase domain protein	YP_821473	−0.898	1.000
von Willebrand factor, type A	YP_821474	−0.583*	1.000
acetolactate synthase, large subunit, biosynthetic type	YP_821479	−1.024	1.00
CnaB-type protein	YP_821495	1.083*	0.140
ASPIC UnbV domain protein	YP_821513	−2.277*	1.000
glycosyl transferase, family 2	YP_821582	−1.069	1.000
NAD-dependent epimerase/dehydratase	YP_821583	−1.561	1.000
aldo/keto reductase	YP_821684	0.914*	0.181
phage integrase family	YP_821644	−1.395*	1.000
phage integrase family	YP_821919	1.873*	0.032
phage integrase family	YP_821920	−0.293	1.000
phage integrase family	YP_821921	−2.001	1.000
integrase, catalytic region	YP_821924	−1.212	1.000
integrase catalytic region	YP_821733	−0.023	1.000
transposase IS3/IS911 family	YP_821734	−0.783	1.000
transposase IS3/IS911 family	YP_821923	−0.824	1.000

*Some of the pairwise comparisons (for examples, see Tables S5, S6, S7) showed significant values (probability less than 0.05, indicating positive selection). These significant values are reflected in higher overall average values of the Z statistic and the lower values of probability. Representative paralog groups were included in this analysis. The identifier of the first sequence is shown in the table, and the remaining paralogs in each group were selected based on the criteria outlined in the methods section. The probability of rejecting the null hypothesis of strict-neutrality (dN = dS) in favor of the alternative hypothesis (dN>dS) is shown (in the probability column). Probability values less than 0.05 are considered significant at the 5% level. The Z statistic (dN - dS) is shown in the Stat column. dS and dN are the numbers of synonymous and nonsynonymous substitutions per site, respectively.

**Table 4:** Results from substitution saturation analysis.

Paralog group (accession of first sequence)	P(invariant)	Saturation test result	Comment
serine/threonine protein kinase (YP_821325)	0.11516	little	
carboxylesterase, type B (YP_821393)	0.01800	little	
acetolactate synthase, large subunit, biosynthetic type (YP_821479)	0.03254	little	
phage integrase family (YP_821919)	0.16027	little	
two component transcriptional regulator (YP_821972)	0.00047	substantial*	
ABC transporter-related (YP_821380)	0.00024	substantial*	
transcriptional repressor, CopY family (YP_821398)	0.00059	substantial*	
anti-sigma factor antagonist (YP_821407)	0.02566	substantial	
RNA polymerase, sigma-24 subunit, ECF subfamily (YP_821437)	0.01145	substantial*	
oxidoreductase domain protein (YP_821473)	0.01711	substantial	
TonB-dependent receptor, plug (YP_821493)	0.00	substantial*	
CnaB-type protein (YP_821495)	0.00	substantial*	
ASPIC/UnbV domain protein (YP_821513)	0.00414	substantial	
glycosyltransferase, family 2 (YP_821582)	0.02855	substantial*	
NAD-dependent epimerase/dehydratase (YP_821583)	0.00208	substantial*	
phage integrase family (YP_821644)	0.00087	substantial*	
aldo/keto reductase (YP_821684)	0.00	substantial*	
drug resistance transporter, EmrB/QacA family (YP_821403)	ND	ND	too few sequences
TonB-dependent receptor, plug (YP_821405)	ND	ND	too divergent
phage tail collar protein (YP_821449)	ND	ND	too few sequences
von Willebrand factor, type A (YP_821474)	ND	ND	too divergent
phage integrase family (YP_821920)	ND	ND	too few sequences
phage integrase family (YP_821921)	ND	ND	too few sequences
transposase, IS3/IS911 family (YP_821923)	ND	ND	too few sequences
integrase catalytic region (YP_821924)	ND	ND	too few sequences
integrase catalytic region (YP_823733)	ND	ND	too few sequences
transposase, IS3/IS911 family (YP_823734)	ND	ND	too few sequences

“little” means that the test showed little substitution saturation in the group of sequences. “substantial” indicates that there was substantial substitution saturation. ND, not determined because there were either too few sequences to test, or the sequences were too divergent.

*indicates sequences that were too divergent to be useful for phylogenetic analyses.

### Acidobacteria genome size survey

**Figure 2:**
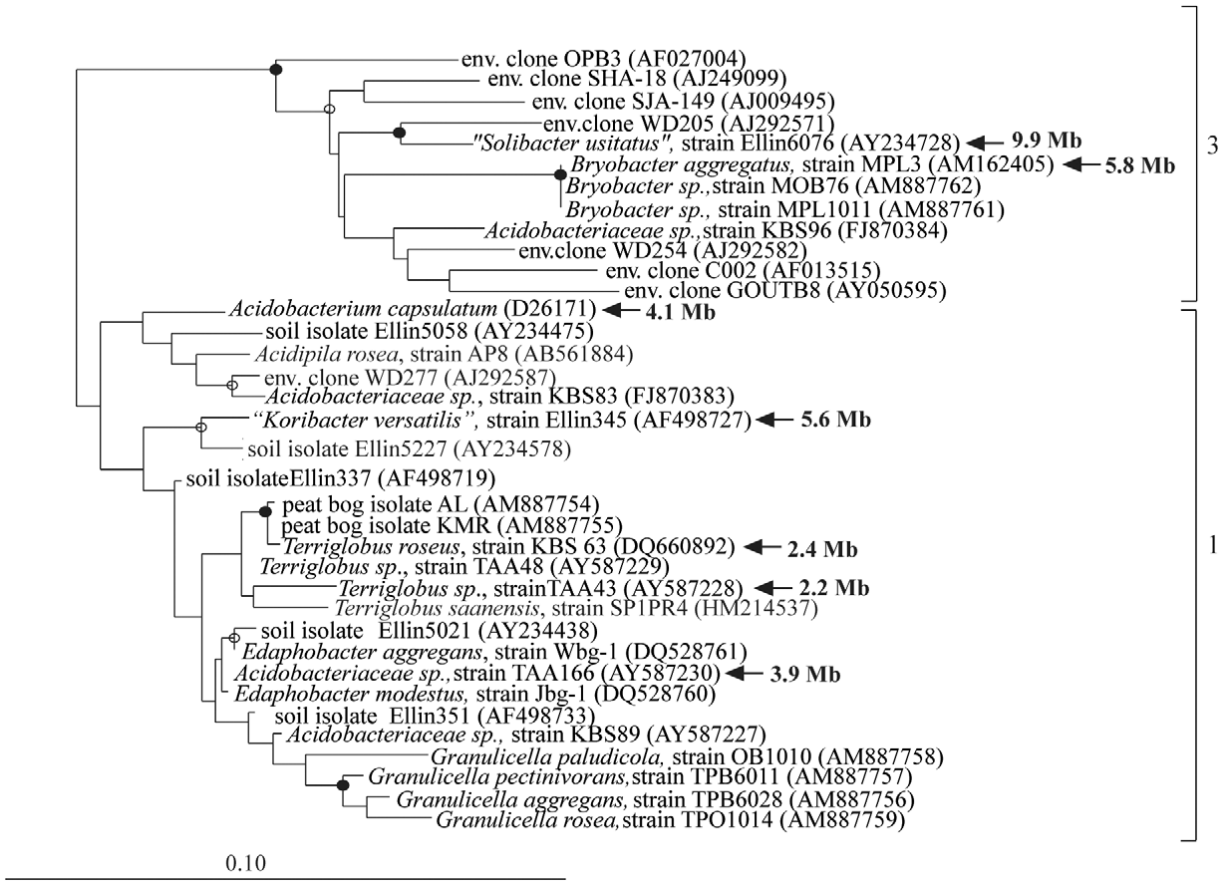
Maximum-likelihood tree of the *Acidobacteria* subdivisions 1 and 3 (indicated to the right of the group) based on the16S rRNA gene using sequences obtained from cultivated representatives and environmental clones. *Geothrix fermentans*, *Holophaga foetida* and *Acanthopleuribacter pedi* of subdivision 8 were used as an outgroup (not shown). Strains for which the genome size has been determined are highlighted in bold typeface. Internal nodes support by a bootstrap value of >95% are indicated with a filled circle and >70% with an open circle. The scale bar indicates 0.10 changes per nucleotide.

Few named and described strains exist to represent the phylum *Acidobacteria*, and most soil isolates represent only two of the twenty-six recognized subdivisions in the phylum [9]. Pulsed-field gel electrophoresis was used to estimate the genome size of other *Acidobacteria* strains from subdivisions 1 and 3 to determine if the large genome trait was specific to Ellin6076 or more widely distributed among the cultured isolates. The pulse field gel sizing of seven *Acidobacteria* strains illustrated that members of subdivision 1 had smaller genomes ranging from 2.0 to 5.7 Mb, and members of subdivision 3 had genomes of 5.8 and 9.9 Mb. (Figure 2, Table S6). In addition, three newly sequenced draft genomes of subdivision 1 strains have estimated genome sizes ranging from 5.1 to 6.2 Mb (*Terriglobus saanensis*, strain SP1PR4, 5.1 Mb; *Acidobacteriaceae* sp., strain MP5ACTX8, 6.2 Mb; *Acidobacteriaceae* sp., strain MP5ACTX9, 5.4 Mb) (http://www.img.jgi.doe.gov).

## Discussion

Our results indicate that multiple mechanisms likely contributed to the large genome of Ellin6076. The lack of strand bias, increased number of repeats, and distribution pattern of the 126 mobile genetic elements throughout the genome of Ellin6076 suggest that horizontal transfer, followed by gene duplication, repeat-mediated recombination and intra-genome transposition may have acted to shape the structure of this genome.

The presence of various phage- and plasmid-related sequences in the Ellin6076 genome indicate that phage- and plasmid-mediated horizontal transfer events did occur, either in this genome or in an ancestral genome. Soils contain abundant and diverse bacteriophage populations [43], so it is reasonable to speculate that soil phage integration contributed to the genome of Ellin6076. However, the genome did not contain intact identifiable prophage regions, but did have numerous mobile genetic elements (i.e. transposases, phage integrases, and IS elements), repeat sequences and scattered phage-related genes. Therefore, while phage integration events likely shaped the Ellin6076 genome, these events were not recent, having been obscured by more recent gene duplications and rearrangements mediated by the abundant mobile elements and repeats. Collectively these events resulted in multiple, divergent paralogs, which provide Ellin6076 with the potential for broader and more differentially regulated metabolic and defensive functions. Adding to the increasing genomic evidence from other bacterial species, our results support the conjecture that large bacterial genomes may result from ancient horizontal transfer events and gene duplication processes [44]. However, proof of this conjecture will require analysis of additional genomes from soil-dwelling bacteria to provide a more detailed understanding of how particular genes and DNA fragments become stabilized in bacterial genomes, and their relationship to an organism's overall fitness with respect to the environment.

Paralogs in the same functional group had relatively low sequence similarities to each other, suggesting that the paralogs in the Ellin6076 genome were produced by ancient horizontal transfer and/or duplication events, followed by mutations that resulted in sequence divergence. To quantify the evolutionary pressures that acted on the paralogous sequences, we performed a codon-based test of positive selection on each of 27 representative paralog groups. Results (Table 3) demonstrated that, while some paralogs within a functional group showed evidence of positive selection, others were either neutral or showed evidence of purifying selection. However, substitution saturation analysis revealed that many of the paralog sequences had experienced saturation, and are therefore too divergent to compare phylogenetically. This further supports our hypothesis that ancient events contributed to the size and structure of the Ellin6076 genome. Although the specific functions of the paralogs in these groups are not known, we can postulate certain activities for these paralogs. For example, bacterial esterases cleave ester bonds of short chain fatty acids [45], and may have diverse functions in the hydrolysis of compounds like beta-lactam antibiotic para-nitrobenzyl esters [46]. CnaB is a repeat-containing domain found in collagen-binding proteins [47], indicating that the CnaB-type protein paralogs may function in binding. It is conceivable that the transporter protein paralogs may have slightly different specificities for the compounds that they transport, adding diversity to the repertoire of drugs that Ellin6076 is able to pump out, or nutrients that it is able to take up via the ABC family of transporters.

Most of the integrase and transposase genes were either identical or nearly identical in sequence, and only one group showed evidence of positive selection (Table 3). Many of the paralog groups contained sequences showing negative values of the Z statistic, and some of these had significant probability values, indicating that they were under purifying selection. This supports our conjecture that at least some of the paralogs may have experienced positive selection, while others are likely in the process of being eliminated through purifying selection.

Examination of the gene neighborhoods surrounding the divergent paralogs revealed the presence of one or more mobile elements (Table S2), indicating the potential for movement within the genome. This observation is consistent with results from studies in cyanobacteria and archaea [48] and *Sulfolobus solfataricus* P2, indicating that IS elements facilitate genomic changes by transposase-mediated transposition and by increases in copy number through repeat-mediated homologous recombination [49] and self-replicating behavior [50]. The number of recently active IS elements increases along with genome size, and the regions adjacent to these IS elements are enriched in genes encoding regulatory and metabolic functions [48]. Consistent with these previous results, we found that COG categories representing regulatory and metabolic functions were expanded in the large genome of Ellin6076 (Table S5), and at least some of them appeared to be stable in the genome. We also identified several groups of related mobile element genes with high sequence similarity (90–100%) to each other (Table S2), located in the vicinity of regulatory and metabolic genes (data not shown), indicating that they may have recently duplicated and moved throughout the genome.

The abundant Ellin6076 paralogs involved in metabolism, defense and regulation, suggest an increased functional diversity in this bacterium. Soil bacteria must cope with extremes of moisture, temperature, and geochemical conditions, and compete successfully with other microbes for limited or rapidly changing nutrient resources. Expansion of the COG functional categories of carbohydrate, amino acid and inorganic transport and metabolism in Ellin6076 (Tables S7, S8, S9) suggests an enhanced competitive ability to exploit different environmental resources. Because Ellin6076 is so challenging to culture, we have not performed detailed metabolic profiling to confirm the potential functions indicated by the gene content. However, some of these traits have previously been verified in culture studies [22], [51]. Carbon utilization analyses of various *Acidobacteria* demonstrate that they are generally able to use simple organic compounds, such as sugars, sugar alcohols and amino acids, as carbon sources for growth. However, the various strains analyzed showed differences with respect to the specific compounds that they could utilize [6], [15], [18], [22], [52]. The diverse array of paralogs in Ellin6076 could confer an expanded set of metabolic and regulatory functions that would be advantageous under widely changing conditions in soil microhabitats. Our finding that paralogs within a particular functional group have divergent sequences compared to each other, and that some of them may have been subjected to positive selection, supports this conjecture. In addition to metabolic exploitation of available resources, Ellin6076's large genome could provide an alternative competitive and survival strategy in adverse environmental conditions, as has been suggested for other soil-dwelling heterotrophs [44], [53]. Functional redundancy in the genome of Ellin6076 could be due to the presence of ecoparalogs [54], which perform the same basic function under different environmental conditions, and can help microorganisms during seasonal periods of fluctuations in resources [55]. Patchy nutrient distribution, limiting nutrients, and geochemical conditions that can vary dramatically across the mm scale, are major factors that shape soil microbial communities [56], [57]. The ability to use varied nutrient sources across gradients of physical conditions would be advantageous in the soil. Ellin6076 has genes involved in cell wall/membrane biogenesis, and numerous paralogs that may function in transcription and signal transduction cascades, suggesting an increased ability to sense and respond to environmental changes, and to regulate metabolism (Table S5). Most notable are genes encoding serine/threonine protein kinases, transcriptional regulators, and DNA-directed RNA polymerase, sigma-24 (sigma E) homologs. To date, the large genome of Ellin6076 contains the most sigma E homologs of any sequenced bacterial genome. Bacterial sigma E regulons are induced in response to stressful environmental conditions including nutrient limitation/starvation, oxidative stress, heat shock, lead exposure, cell envelope stress [58], [59], [60], [61], [62], [63], [64]. Processes activated by sigma E include outer membrane synthesis and assembly [65], carotenoid biosynthesis [66], mucoid production [67], and organism-specific functions necessary for environmental adaptation [65]. Ellin6076's increased capacity to respond to the environment may provide a selective advantage in times of stress.

The functional complexity gained with a large genome may not be unique to soil microorganisms. Increased bacterial genome size (6 Mb) has also been observed at the border of the oxic and anoxic zones in microbial mats, compared to the rest of the mat (3–3.5 Mb) possibly reflecting an increased functional complexity needed to survive and thrive at this depth [68]. The large (7.2 Mb) genome of the marine bacterium *Hahella chejuensis* also has a number of functionally redundant genes involved in transcriptional regulation and/or environmental sensing that may play roles in its adaptability to a changing marine environment [69].

Currently, the large genome of Ellin6076 is unique among the few cultured *Acidobacteria* for which we have estimated genome sizes. However, the Ellin6076 and Ellin345 pair represent two different subdivisions within the phylum (Figure 2), and are not as closely related as the other pairs in Table 1. The large genome trait occurs sporadically among closely related species, as evidenced by studies of genomes greater than 6 Mb in size such as *Hahella chejuensis* KCTC 2396 [69], *Bradyrhizobium japonicum* USDA 110 [70], *Mesorhizobium loti* MAFF303099 [71], *Streptomyces coelicolor* A3(2) [24], *Streptomyces avermitilis* MA-4680 [72], *Rhodococcus* sp. RHA1 [44], and *Burkholderia xenovorans* LB400 [53]. Like Ellin6976, these large genomes also demonstrated increased numbers of paralogs (Tables 1 and S4). A previous study quantified genome size and paralog numbers within 106 complete bacterial genomes, showing that size was strongly correlated with the number of paralogs, which represented functional classes of genes involved in adaption to the environment [33].

In conclusion, our results indicate that the large genome of Ellin6076 has arisen through horizontal gene transfer via ancient bacteriophage and plasmid-mediated transduction, as well as widespread small-scale gene duplications, resulting in an increased number of paralogs. The low amino acid sequence identities, and correspondingly divergent nucleotide sequences, among paralogs encoding similar functions argue against recent duplication events. Ellin6076 appears to be ancient, and the abundant paralogs encode traits that may provide a variety of metabolic, defensive and regulatory functions in the soil environment. The large genome of Ellin6076, along with improved culture approaches and studies of the *Acidobacteria* in soil, will facilitate future biological and physiological studies to ultimately determine the costs and benefits of harboring a large genome.

## Materials and Methods

### Genome sequencing and annotation

Genome sequencing and annotation were described previously [22].

### Computational analysis

The MUMmer package [34] programs nucmer, repeat_match and exact_tandems were used for analysis of repeat regions. To identify long inexact genomic repeats, the nucmer program was used with the options –maxmatch and –nosimplify to align the Ellin6076 genome against itself. Dotplots were generated by mummerplot. Smaller genomic repeats were identified using repeat-match (finds all repeats), exact-tandems (exact tandem repeats), Tandem Repeats Finder (all tandem repeats, [73]), and Inverted Repeats Finder (inverted repeats, [74]). The repeat_match and exact_tandems programs were run with default arguments. Tandem repeats finder [73] and inverted repeats finder [74] were run with recommended default arguments to identify tandem and inverted repeats, respectively.

Distributions of paralogs and orthologs in the Clusters of Orthologous Groups of proteins (COGs) categories [75] were obtained from the Integrated Microbial Genomes (IMG) system (http://img.doe.gov), using Reverse Position Specific BLAST of the Ellin6076 sequences against NCBI's Conserved Domain Database as described in (http://img.jgi.doe.gov/w/doc/userGuide.pdf). Pairwise relationships were computed as reciprocal hits within the genome, and paralogous groups were identified using the Markov Cluster Algorithm (MCL) with default parameters.

Comparative genome analyses were performed using the IMG system [76], BLAST [77], the Conserved Domain Database [75], MetaCyc (http://metacyc.org) and output from the Pathway Tools [78]. The cumulative GC skew was determined as described in [79] using the GenSkew application (http://genskew.csb.univie.ac.at/). Putative genomic islands were identified and analyzed using the IslandPath [35] web resource (http://www.pathogenomics.sfu.ca/islandpath/cgi-bin/islandupdate.pl?gc=Submit&organism=Sousi008536). The IMG system was used to identify clustered regularly interspaced short palindromic repeats (CRISPRs)[76].

### Identification of paralogous gene families

Paralogs were identified by BLAST sequence similarity comparisons of the collection of Ellin6076 protein sequences against each other using a threshold E-value of 1.00E-05. Paralogs were grouped through manual examination of the tabular formatted BLAST results; sequences were included in a particular paralog group only if the alignment to the query sequence covered at least 90% of the query sequence length. Phylogenetic analysis was performed for 27 representative paralog groups, using the Phylogeny.fr web service [80] and MEGA5 [81]. For each paralog group, the “One Click” pipeline at Phylogeny.fr was used, consisting of MUSCLE sequence alignment, Gblocks alignment curation, PhyML phylogenetic analysis and TreeDyn tree rendering. Within this pipeline, MUSCLE was used in full processing mode. Gblock settings were: Min. seq. for flank pos.: 85%; Max. contig. nonconserved pos.: 8; Min. block length: 10; Gaps in final blocks: no. PhyML was used with the aLRT statistical test. The PhyML settings were: Model: Default; Statistical test: alrt; Number of categories: 4; Gamma: estimated; Invariable sites: estimated; Remove gaps: enabled. TreeDyn tree rendering settings were: Conformation: rectangular; Legend: displayed; Branch annotation: bootstrap; Font: Times 8 normal. The codon-based Z-test of positive selection was performed on each paralog group using MEGA5 [81]. Analyses were conducted using MUSCLE for sequence alignments and the Nei-Gojobori substitution model/method [42]. All positions containing gaps and missing data were eliminated. The codon-based Z-test compares the number of non-synonymous mutations that would lead to a change in the translated protein sequence, with synonymous mutations that are neutral and do not change the protein sequence. The codon-based Z-test was performed on each pair of sequences within each paralog group, as well as all sequences in the group to obtain an average number of nonsynonymous substitutions. For each pair of sequences, MEGA5 estimates the number of synonymous substitutions per synonymous site (*d*_S_) and the number of nonsynonymous substitutions per nonsynonymous site (*d*_N_), and their variances: Var(*d*_S_) and Var(*d*_N_), respectively. With this information, we used the MEGA5 package to test the hypothesis that H_0_: *d*_N_>*d*_S_ (positive selection) using a one-tailed Z-test: Z =  (*d*_N_–*d*_S_) / SQRT(Var(*d*_S_) + Var(*d*_N_)) [81]. The variance of the difference was computed using the bootstrap resampling method (500 replicates). We also used MEGA5 to compute the average number of synonymous substitutions and the average number of nonsynonymous substitutions to conduct a Z-test in a manner similar to the pairwise test described above. The variance of the difference between these two quantities was also estimated by the bootstrap resampling method. If the number of non-synonymous mutations is greater than the synonymous mutations, the value of the test statistic Z is greater than one, and a corresponding probability of less than 0.05 is evidence for positive selection (or when a non-synonymous mutation becomes fixed in the genome). A Z value of less than one provides evidence of purifying selection, or selection against deleterious amino acid changes [42]. To analyze potential substitution saturation, which would overwrite past changes in the paralog nucleotide sequences, we performed a substitution saturation test using DAMBE [82]. For each paralog group, this test involved estimating the proportion of invariant sites among the sequences, and using this proportion in a test of substitution saturation [83].

### 16S rRNA phylogenetic tree generation

Sequences were aligned using the SILVA [12] website and the phylogenetic tree was generated in the ARB Software [84]. The maximum likelihood algorithm (RAxML) in ARB was used for the generation of the phylogenetic trees with a base frequency filter with a minimum and maximum sequence similarity of 70% and 100%, respectively. The filter was designed from nearly full-length, high quality acidobacteria sequences across the subdivisions. Bootstrapping was done in the ARB software using the rapid bootstrap analysis with 100 iterations.

### Genome size determination

The genome sizes of seven subdivision 1 and 3 *Acidobacteria* strains (Table S6) were estimated using pulsed-field gel electrophoresis after restriction of genomic DNA using *SwaI* and *PmeI* enzymes (New England Biolabs, Beverly, MA) as described previously [85]. Restricted genomic DNA was separated by electrophoresis on a CHEF-DR apparatus (Bio-Rad Laboratories, Richmond, CA), together with yeast chromosome, Lambda ladder, and low range molecular size markers (New England Biolabs, Beverely, MA). The isolates represent different genera, with 16S rRNA gene sequence similarity (a measure of bacterial relatedness) of ca. 90% between the subdivision 3 strains (n = 2), and of ca. 92% among the subdivision 1 strains (n = 5: Figure 2).

### Data availability

The genome sequences of Ellin6076 and Ellin345 are in GenBank (NC_008536, NC_008009).

## Supporting Information

Figure S1 **Dotplot showing the Ellin6076 genome nucleotide sequence aligned against itself.** The alignment and dotplot were generated by the MUMmer package programs nucmer (using arguments -maxmatch –nosimplify) and mummerplot.
(PDF)

Figure S2 **Cumulative GC-skew plots for** ***Candidatus*** **Solibacter usitatus Ellin6076 (panel A) and** ***Candidatus*** **Korebacter versatilis Ellin345 (panel B).** Plots were generated with the GenSkew application (http://genskew.csb.univie.ac.at/).
(PDF)

Figure S3 **Phylogenetic trees showing the relationships of the CnaB-type protein (panel A) and oxidoreductase domain protein (panel B) paralogs to each other.** Trees were generated using the Phylogeny.fr web service (http://www.phylogeny.fr).
(PDF)

Figure S4 **Phylogenetic trees showing the relationships of the serine/threonine protein kinase (panel A), Drug resistance transporter, EmrB/QacA subfamily (panel B), glycosyl transferase family protein (panel C), acetolactate synthase, large subunit (panel D), and RNA polymerase, sigma-24 subunit, ECF subfamily (panel E) paralogs to each other.** Trees were generated using the Phylogeny.fr web service (http://www.phylogeny.fr).
(PDF)

Figure S5 **Results of pairwise codon-based test of positive selection for CnaB-type protein (YP_821495.1) paralogs.**
(TIFF)

Figure S6 **Results of pairwise codon-based test of positive selection for phage tail collar protein (YP_821449.1) paralogs.** Sequence #3 was included twice (as sequence #4) to show the probability for identical sequences.
(TIFF)

Figure S7 **Results of pairwise codon-based test of positive selection for phage integrase (YP_821919.1) paralogs.**
(TIFF)

Table S1 **Total number of repeats in the Ellin6076 genome compared to Ellin345.**
(DOC)

Table S2 **Ellin6076 mobile elements.**
(DOC)

Table S3 **Candidate genomic islands in the Ellin6076 genome.**
(DOC)

Table S4 **Distribution of genes in COG categories for** ***Acidobacteria*** **strains Ellin6076 and Ellin345, compared to other large-small genome pairs.**
(DOC)

Table S5 **Distribution of genes in COG categories for** ***Acidobacteria*** **strains Ellin6076 and Ellin345.**
(DOC)

Table S6 **Average genome size of representative** ***Acidobacteria*** **isolates from subdivisions 1 and 3, determined by pulse field gel electrophoresis.**
(DOC)

Table S7 **Expansion of COG categories for carbohydrate transport and metabolism.**
(DOC)

Table S8 **Expansion of COG categories for amino acid transport and metabolism.**
(DOC)

Table S9 **Expansion of COG categories for inorganic transport and metabolism.**
(DOC)
