# Supplementary figures and images for: Biological Consequences of Ancient Gene Acquisition and Duplication in the Large Genome of Candidatus Solibacter usitatus Ellin6076
Source: PLoS One. 2011 Sep 15;6(9):e24882. doi: 10.1371/journal.pone.0024882 (PMC3174227; doi:10.1371/journal.pone.0024882)

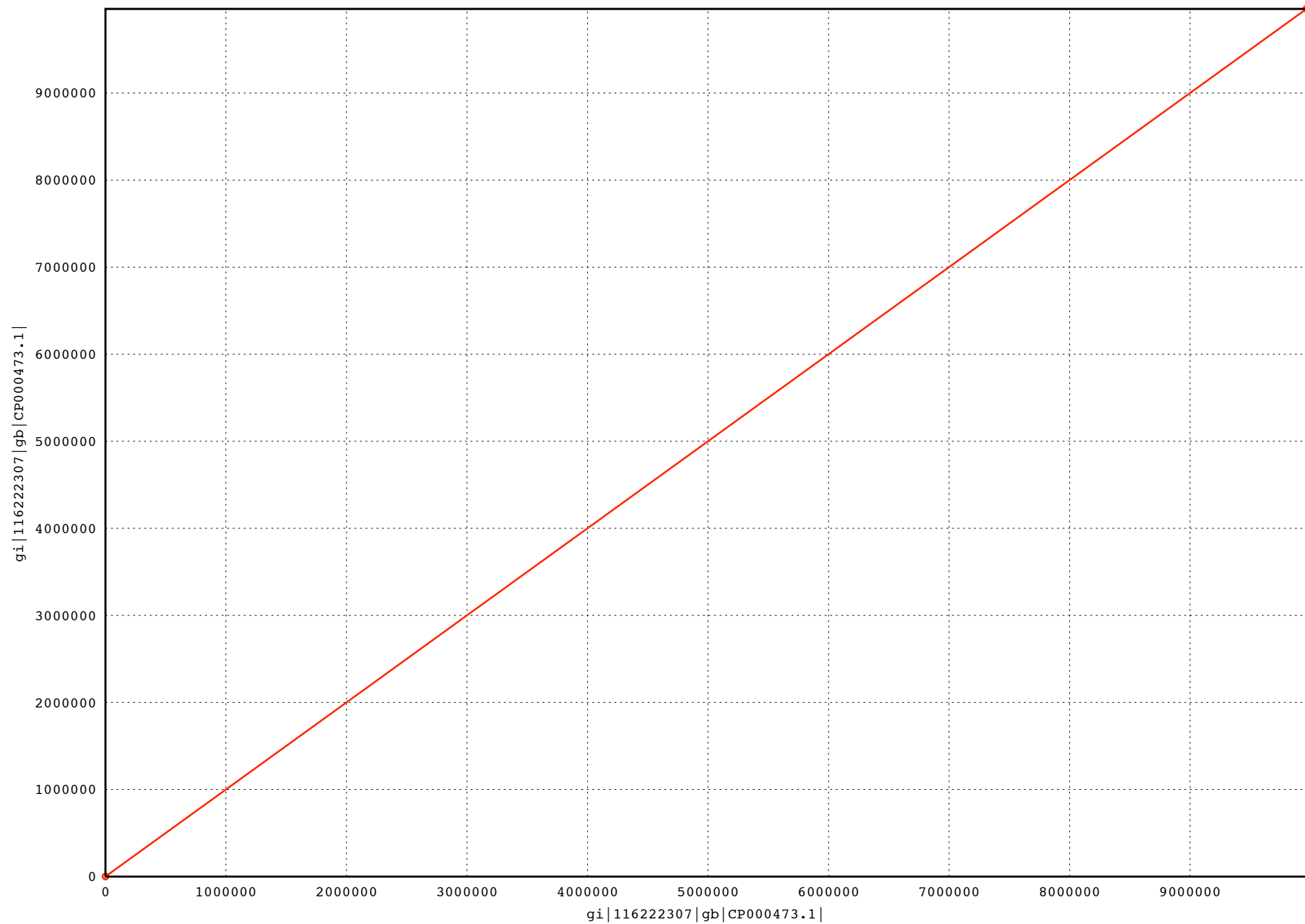

Supplement: Figure S1 — Dotplot showing the Ellin6076 genome nucleotide sequence aligned against itself. The alignment and dotplot were generated by the MUMmer package programs nucmer (using arguments -maxmatch –nosimplify) and mummerplot. (PDF) [file pone.0024882.s001.pdf]

## A. CnaB-type protein

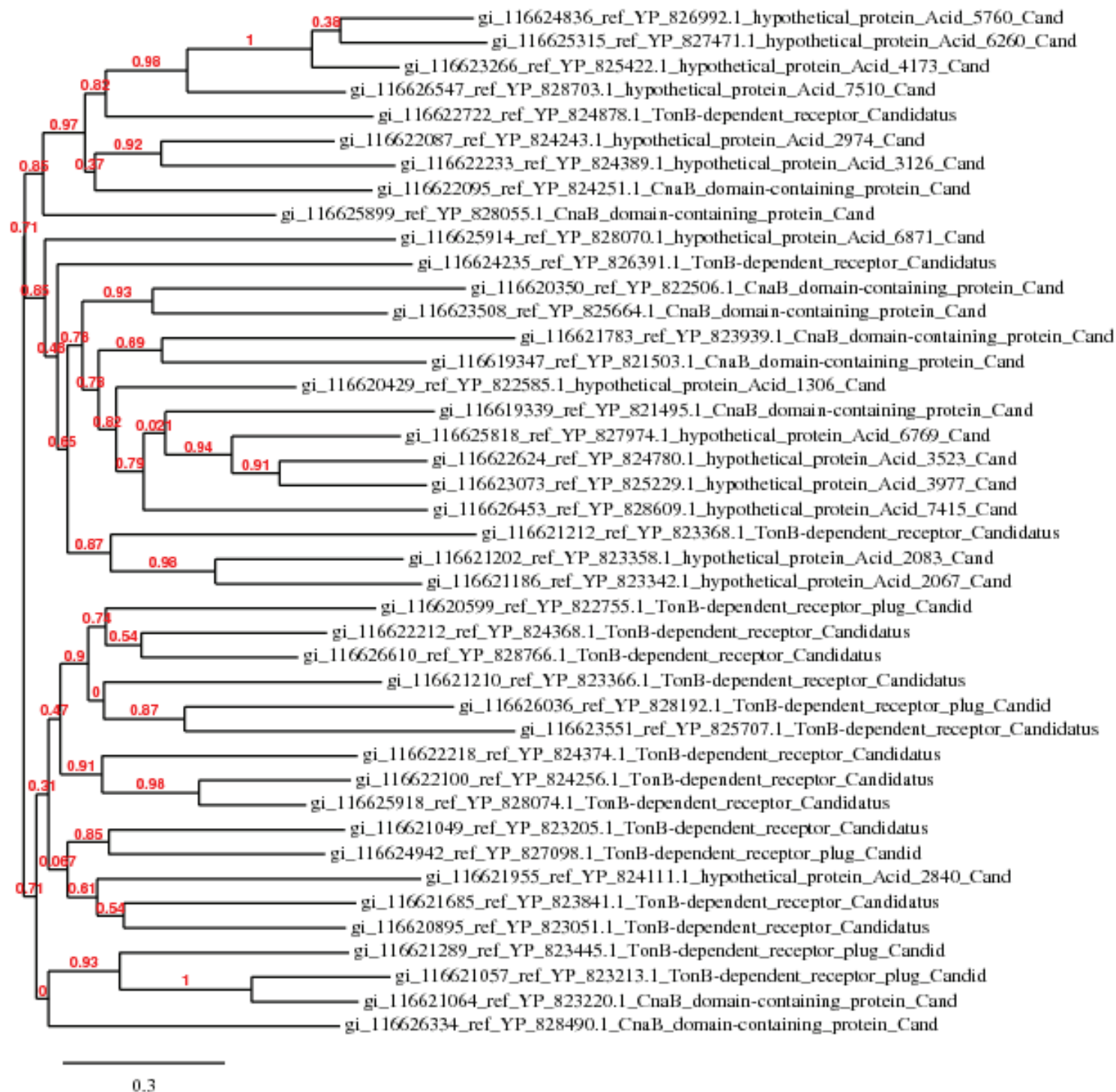

## B. oxidoreductase domain protein

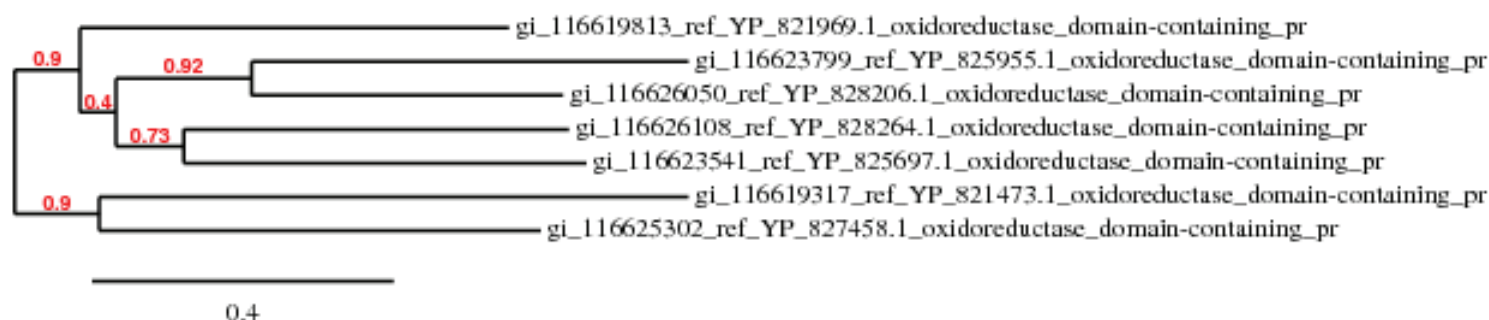

Supplement: Figure S3 — Phylogenetic trees showing the relationships of the CnaB-type protein (panel A) and oxidoreductase domain protein (panel B) paralogs to each other. Trees were generated using the Phylogeny.fr web service (http://www.phylogeny.fr). (PDF) [file pone.0024882.s003.pdf]

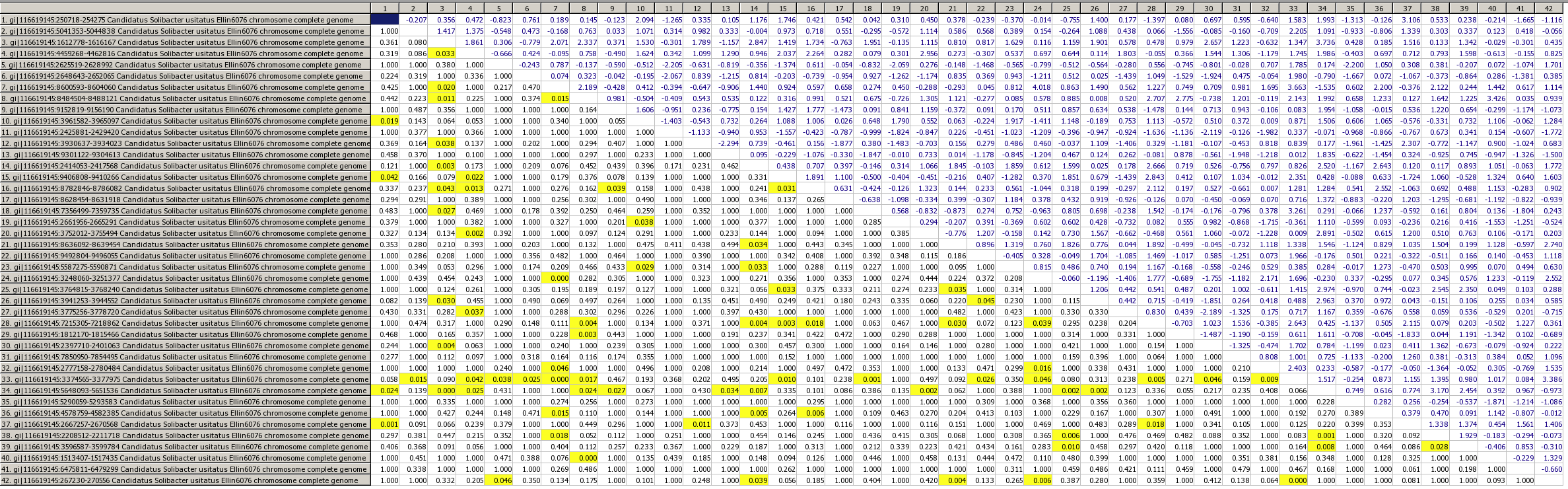

Supplement: Figure S5 — Results of pairwise codon-based test of positive selection for CnaB-type protein (YP_821495.1) paralogs. (TIFF) [file pone.0024882.s005.tiff]

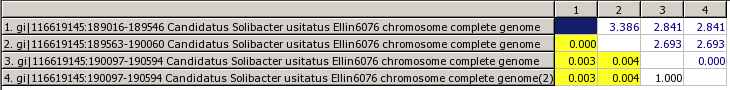

Supplement: Figure S6 — Results of pairwise codon-based test of positive selection for phage tail collar protein (YP_821449.1) paralogs. Sequence #3 was included twice (as sequence #4) to show the probability for identical sequences. (TIFF) [file pone.0024882.s006.tiff]

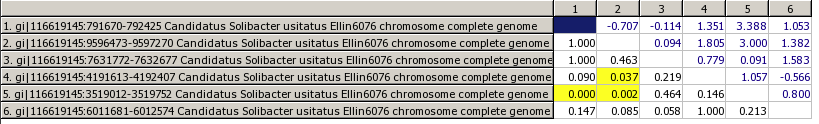

Supplement: Figure S7 — Results of pairwise codon-based test of positive selection for phage integrase (YP_821919.1) paralogs. (TIFF) [file pone.0024882.s007.tiff]
