# Supplementary material for: Biological Consequences of Ancient Gene Acquisition and Duplication in the Large Genome of Candidatus Solibacter usitatus Ellin6076
Source: PLoS One. 2011 Sep 15;6(9):e24882. doi: 10.1371/journal.pone.0024882 (PMC3174227; doi:10.1371/journal.pone.0024882)
Supplement: Figure S2 — Cumulative GC-skew plots for Candidatus Solibacter usitatus Ellin6076 (panel A) and Candidatus Korebacter versatilis Ellin345 (panel B). Plots were generated with the GenSkew application (http://genskew.csb.univie.ac.at/). (PDF) [file pone.0024882.s002.pdf]

A.

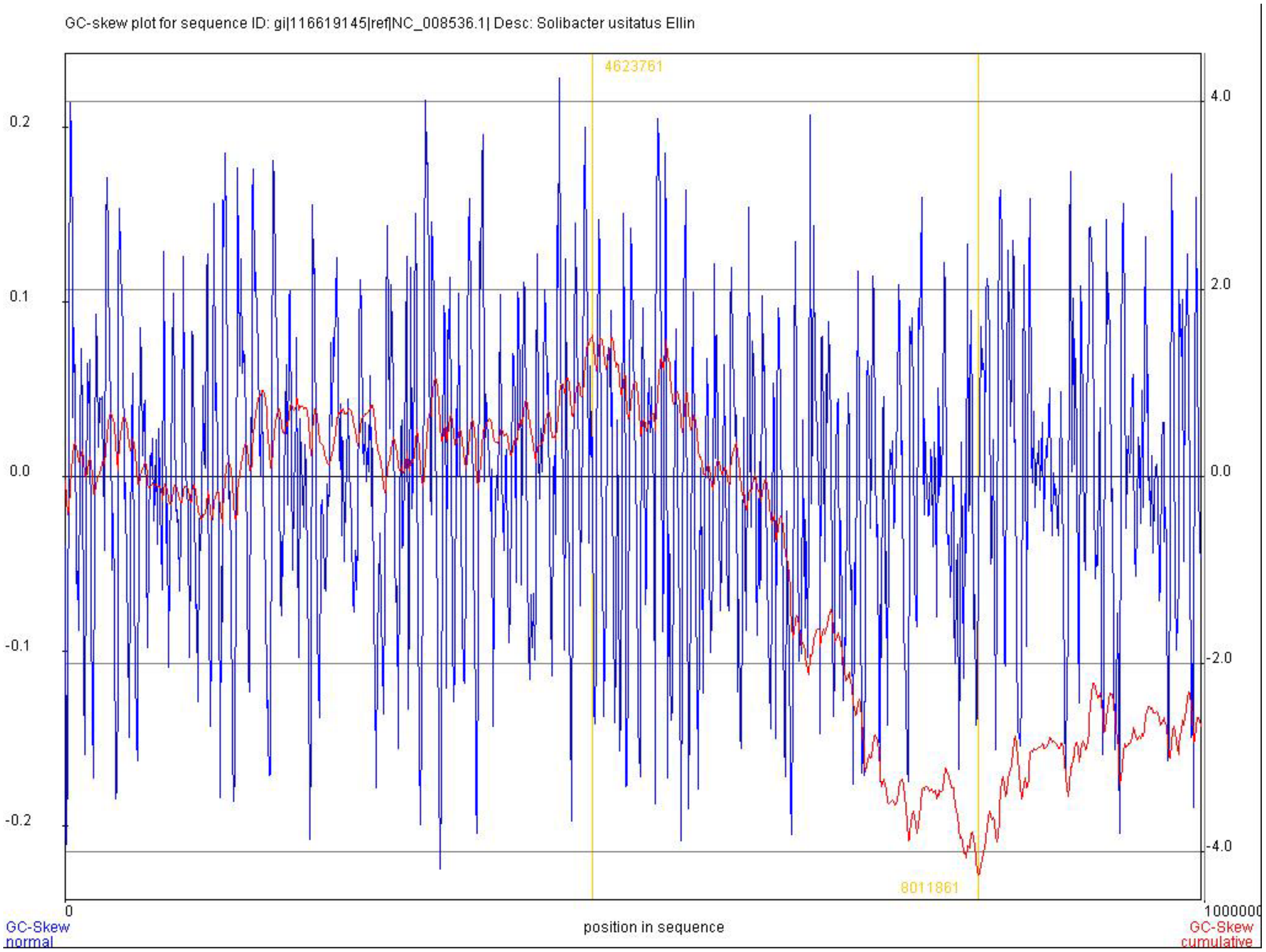
GC-skew plot for sequence ID: gi|116619145|ref|NC_008536.1| Desc: Solibacter usitatus Ellin
4623761
8011861
0.2
0.1
0.0
-0.1
-0.2
4.0
2.0
0.0
-2.0
-4.0
0
1000000
position in sequence
GC-Skew normal
GC-Skew cumulative

B.

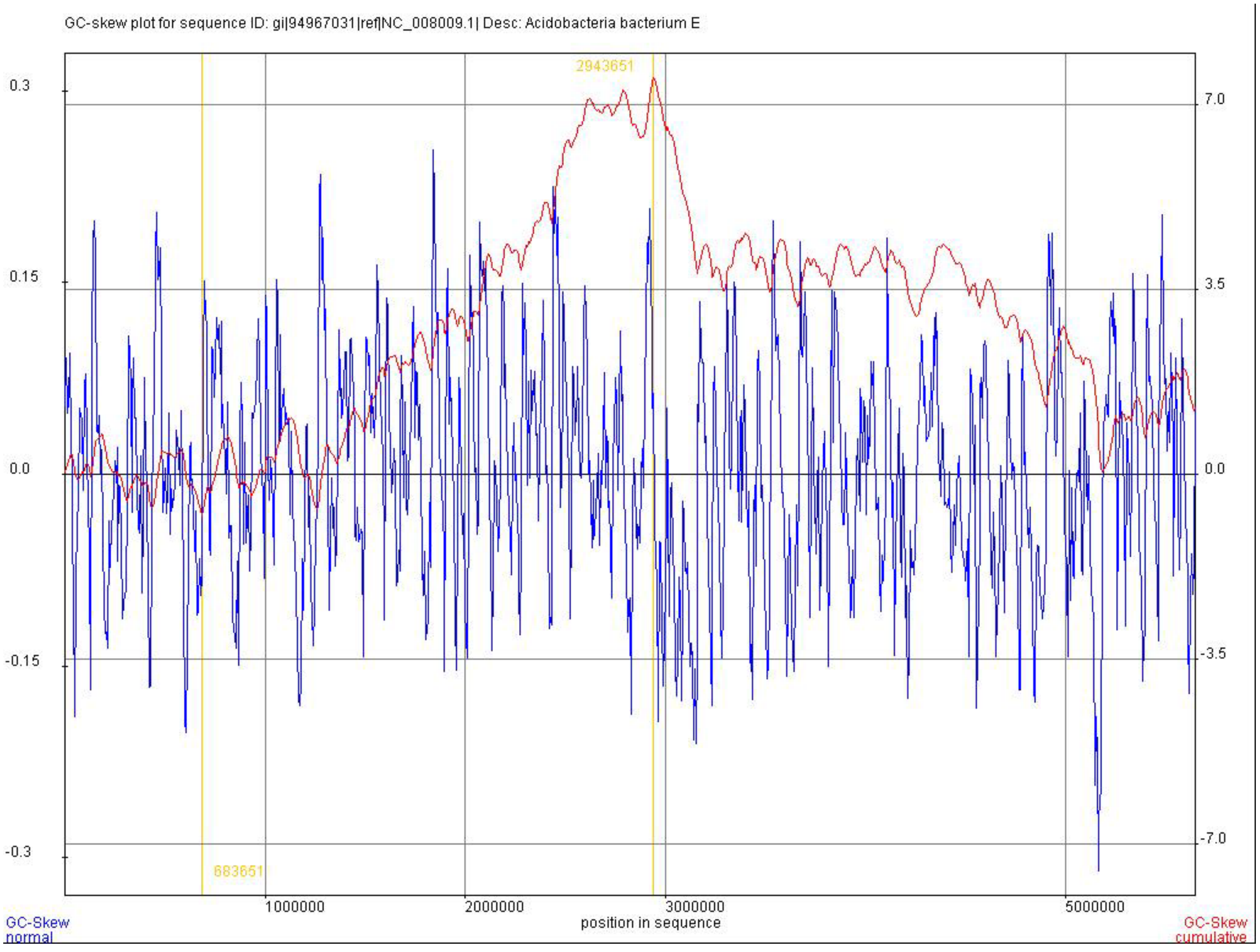
GC-skew plot for sequence ID: gi|94967031|ref|NC_008009.1| Desc: Acidobacteria bacterium E
2943651
683651
0.3
0.15
0.0
-0.15
-0.3
7.0
3.5
0.0
-3.5
-7.0
1000000
2000000
3000000
5000000
position in sequence
GC-Skew normal
GC-Skew cumulative
