# Supplementary material for: Biological Consequences of Ancient Gene Acquisition and Duplication in the Large Genome of Candidatus Solibacter usitatus Ellin6076
Source: PLoS One. 2011 Sep 15;6(9):e24882. doi: 10.1371/journal.pone.0024882 (PMC3174227; doi:10.1371/journal.pone.0024882)

A. serine/threonine protein kinase

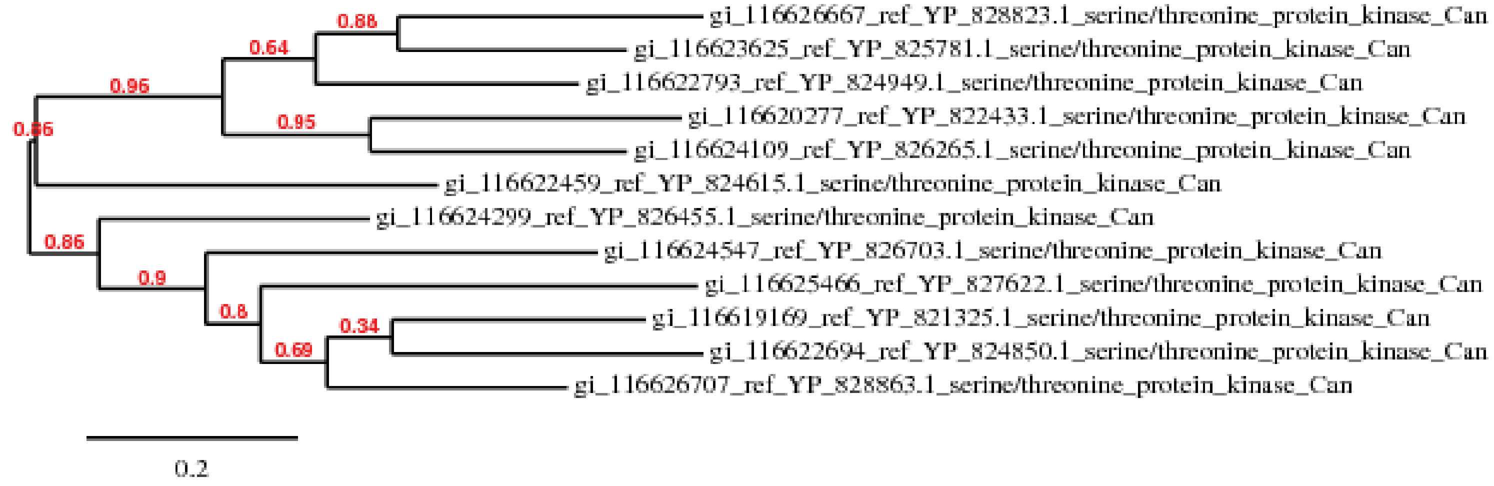

B. Drug resistance transporter, EmrB/QacA subfamily

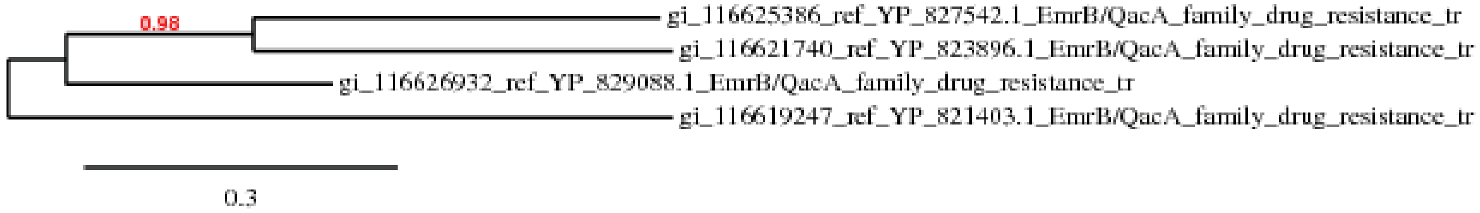

C. glycosyl transferase family protein

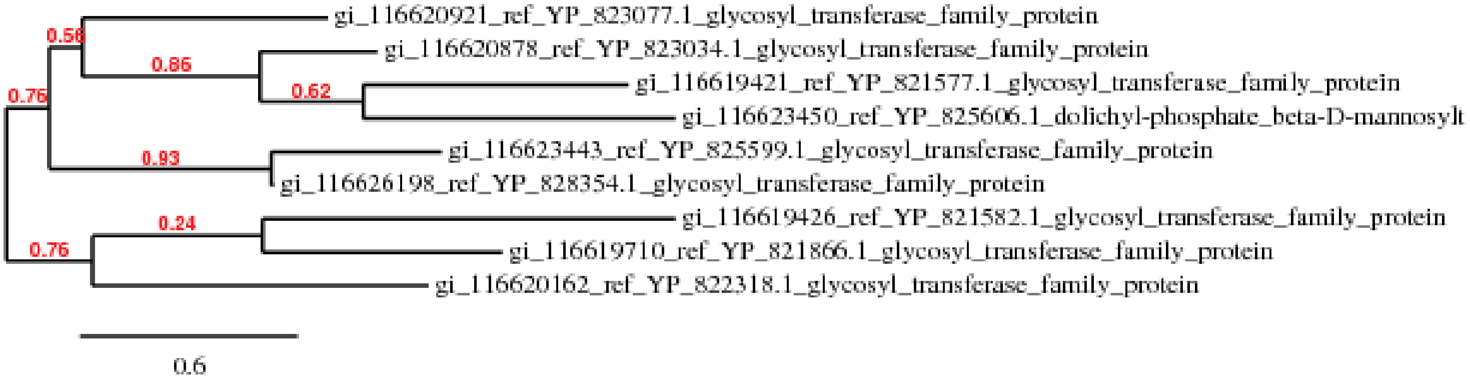

D. acetolactate synthase, large subunit

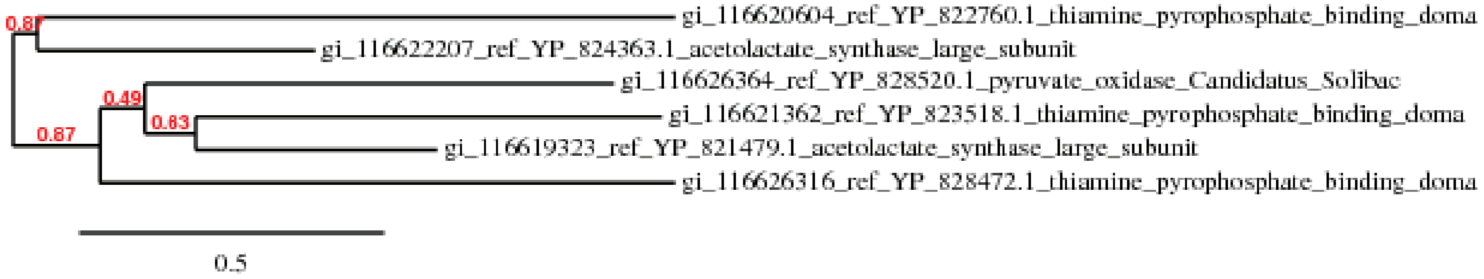

E. RNA polymerase, sigma-24 subunit, ECF subfamily

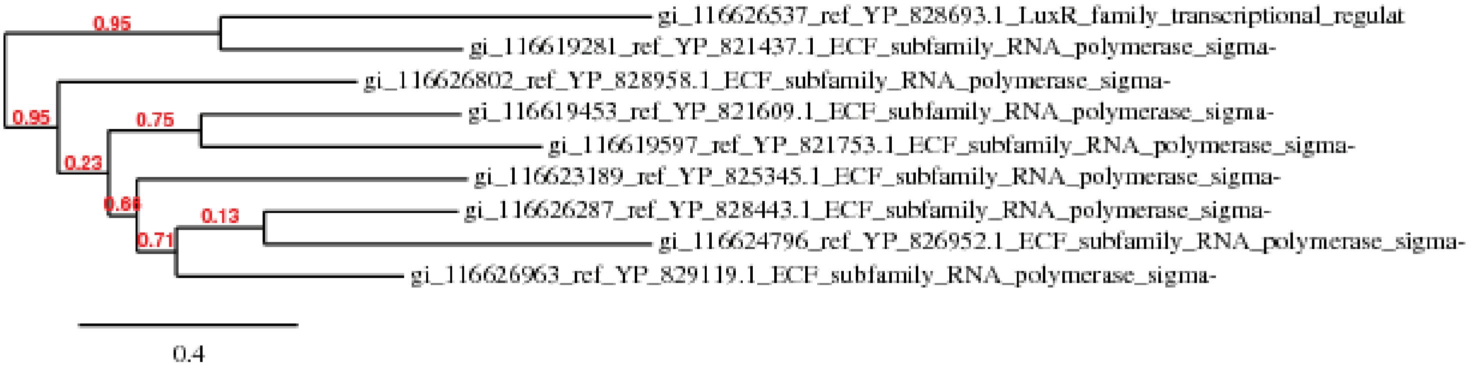

Supplement: Figure S4 — Phylogenetic trees showing the relationships of the serine/threonine protein kinase (panel A), Drug resistance transporter, EmrB/QacA subfamily (panel B), glycosyl transferase family protein (panel C), acetolactate synthase, large subunit (panel D), and RNA polymerase, sigma-24 subunit, ECF subfamily (panel E) paralogs to each other. Trees were generated using the Phylogeny.fr web service (http://www.phylogeny.fr). (PDF) [file pone.0024882.s004.pdf]
