# Supplementary material for: Biological Consequences of Ancient Gene Acquisition and Duplication in the Large Genome of Candidatus Solibacter usitatus Ellin6076
Source: PLoS One. 2011 Sep 15;6(9):e24882. doi: 10.1371/journal.pone.0024882 (PMC3174227; doi:10.1371/journal.pone.0024882)
Supplement: Table S1 — Total number of repeats in the Ellin6076 genome compared to Ellin345. (DOC) [file pone.0024882.s008.doc]

**Table S1.** Total number of repeats in the Ellin6076 genome compared to Ellin345

| Program | No. in Ellin6076 (size range) | No. in Ellin345 (size range) |
| --- | --- | --- |
| repeat-match | 63,764 (20 – 3027) | 7294 (20 – 2525) |
| exact-tandems | 31 (3 – 261) | 19 (7 – 100) |
| Tandem Repeats Finder | 302 (3 – 417) | 154 (6 – 279) |
| Inverted Repeats Finder | 120 (23 – 4436) | 34 (21 – 3018) |
