# Supplementary material for: Biological Consequences of Ancient Gene Acquisition and Duplication in the Large Genome of Candidatus Solibacter usitatus Ellin6076
Source: PLoS One. 2011 Sep 15;6(9):e24882. doi: 10.1371/journal.pone.0024882 (PMC3174227; doi:10.1371/journal.pone.0024882)
Supplement: Table S2 — Ellin6076 mobile elements. (DOC) [file pone.0024882.s009.doc]

**Table S2.** Ellin6076 mobile elements

| Type | Locus_tag | Domains from NCBI Conserved Domain Search | Identity group |
| --- | --- | --- | --- |
| phage integrase family protein | Acid_0347 | INT_XerDC  COG4974 - Site-specific recombinase XerD | none |
| phage integrase family protein | Acid_0629 | INT_Int1 | 5 |
| phage integrase family protein | Acid_0630 | INT_pAE1  Phage integrase, N-terminal SAM-like domain.  COG4974 - Site-specific recombinase XerD | 9 |
| phage integrase family protein | Acid_0632 | INT_SG5  COG4974 - Site-specific recombinase XerD | 10 |
| phage integrase family protein | Acid_0633 | INT_pAE1  COG4974 - Site-specific recombinase XerD | none |
| transposase IS3/IS911 family protein | Acid_0634 | COG2963 | 1 |
| integrase catalytic region | Acid_0635 | pfam00665 rve | 1 |
| Transposase, IS204/IS1001/IS1096/IS1165 family | Acid_0644 | COG3464 | none |
| integrase catalytic region | Acid_0719 | pfam00665 rve  HTH_7 | 6 |
| integrase catalytic region | Acid_0819 | pfam00665 rve  pfam08281 Sigma70_r4_2 | 6 |
| integrase catalytic region | Acid_0942 | pfam00665  rve | 2 |
| transposase IS3/IS911 family | Acid_0943 | pfam01527 transposase_8 | 2 |
| integrase catalytic region | Acid_1129 | pfam00665 rve  HTH_7 | 6 |
| transposase IS116/IS110/IS902 family | Acid_1319 | pfam01548 Transposase_9 pfam02371  transposase_IS116/IS110/IS902 | 7 |
| integrase catalytic region | Acid_1329 | pfam00665 rve  HTH_7 | 6 |
| transposase IS3/IS911 family | Acid_1503 | Pfam01527 transposase_8 | 3 |
| integrase catalytic region | Acid_1504 | pfam00665 rve | 3 |
| integrase catalytic region | Acid_1623 | pfam00665 rve | none |
| transposase IS3/IS911 family | Acid_1624 | COG2963 | 1 |
| integrase catalytic region | Acid_1625 | pfam00665  rve | 1 |
| putative transposase protein Y4bF | Acid_1944 | no domains defined | 8 |
| putative transposase protein Y4bF | Acid_1955 | 100% identical to Acid_1944  no domains defined | 8 |
| integrase catalytic region | Acid_2150 | pfam00665 rve  HTH_7 | 6 |
| transposase IS116/IS110/IS902 family | Acid_2198 | Pfam02371 Transposase IS116/IS110/IS902 family | 11 |
| integrase catalytic region | Acid_2230 | pfam00665 rve  HTH_7 | 6 |
| integrase catalytic region | Acid_2273 | pfam00665 rve | 2 |
| transposase IS3/IS911 family | Acid_2274 | Pfam01527 transposase_8 | 2 |
| integrase catalytic region | Acid_2459 | pfam00665 rve | 2 |
| transposase IS3/IS911 family | Acid_2460 | Pfam01527 transposase_8 | 2 |
| phage integrase family | Acid_2520 | INT_pAE1 | none |
| phage integrase family | Acid_2521 | INT_SG4  COG4974 - Site-specific recombinase XerD | none |
| phage integrase family | Acid_2522 | INT_SG5  COG4974 - Site-specific recombinase XerD | none |
| transposase IS66 | Acid_2528 | pfam03050 | none |
| transposase IS3/IS911 family | Acid_2592 | Pfam01527 transposase_8 | 3 |
| integrase catalytic region | Acid_2593 | pfam00665  rve | 3 |
| transposase IS116/IS110/IS902 family | Acid_2663 | pfam01548 Transposase_9 pfam02371 Transposase_20  transposase_IS116/IS110/IS902 | 7 |
| transposase IS116/IS110/IS902 family | Acid_2712 | pfam01548 Transposase_9 pfam02371 Transposase_20  transposase_IS116/IS110/IS902 | 7 |
| phage integrase family protein | Acid_2776 | INT_Int1  COG4974 - Site-specific recombinase XerD | 4 |
| putative transposase | Acid_2778 | pfam04986 - Transposase_32  IPR007069  Transposase, IS801/IS1294 | 4 |
| transposase IS116/IS110/IS902 family | Acid_2878 | pfam01548 Transposase_9 pfam02371 Transposase_20  transposase_IS116/IS110/IS902 | 7 |
| phage integrase family protein | Acid_2897 | INT_phiLC3_C  Pfam01935 DUF87  Pfam00589? | none |
| transposase IS66 | Acid_2942 | pfam03050 Transposase IS66 family | 12 |
| phage integrase family protein | Acid_2943 | INT_SG5  COG4974 - Site-specific recombinase XerD | none |
| phage integrase family protein | Acid_2945 | INT_pAE1  COG4974 - Site-specific recombinase XerD | none |
| transposase IS66 | Acid_2946 | no conserved domains | none |
| transposase IS3/IS911 family | Acid_3155 | Pfam01527 transposase_8 | 2 |
| integrase catalytic region | Acid_3156 | pfam00665  rve | 2 |
| transposase IS116/IS110/IS902 family | Acid_3180 | pfam02371 Transposase_20  transposase_IS116/IS110/IS902 | 13 |
| putative transposase protein Y4bF | Acid_3260 | no domains defined  100% identical to the other Y4bF genes | 8 |
| putative transposase | Acid_3308 | pfam04986 - Transposase_32  IPR007069  Transposase, IS801/IS1294 | 4 |
| phage integrase family protein | Acid_3310 | INT_pAE1  Phage integrase, N-terminal SAM-like domain.  COG4974 - Site-specific recombinase XerD | 9 |
| phage integrase family protein | Acid_3311 | INT_SG4  COG4974 - Site-specific recombinase XerD | 14 |
| phage integrase family protein | Acid_3312 | INT_SG5  COG4974 - Site-specific recombinase XerD | 10 |
| phage integrase family protein | Acid_3313 | INT_Int1  COG4974 - Site-specific recombinase XerD | 4 |
| integrase catalytic region | Acid_3574 | pfam00665 rve | 2 |
| transposase IS3/IS911 family | Acid_3575 | Pfam01527 transposase_8 | 2 |
| integrase catalytic region | Acid_3877 | pfam00665 rve  HTH_7 | 6 |
| transposase IS116/IS110/IS902 family | Acid_3943 | pfam01548 Transposase_9 pfam02371 Transposase_20  transposase_IS116/IS110/IS902 | 7 |
| transposase IS116/IS110/IS902 family | Acid_3955 | pfam01548 Transposase_9 pfam02371 Transposase_20  transposase_IS116/IS110/IS902 | 7 |
| transposase IS116/IS110/IS902 family | Acid_4031 | pfam01548 Transposase_9 pfam02371 Transposase_20  transposase_IS116/IS110/IS902 | 7 |
| transposase IS116/IS110/IS902 family | Acid_4465 | pfam01548 Transposase_9 pfam02371 Transposase_20  transposase_IS116/IS110/IS902 | 13 |
| integrase catalytic region | Acid_4625 | pfam00665 rve  HTH_7 | 6 |
| phage integrase family | Acid_4695 | INT_P4 | none |
| integrase catalytic region | Acid_4733 | pfam00665 rve | 3 |
| transposase IS3/IS911 family | Acid_4734 | Pfam01527 transposase_8 | 3 |
| transposase | Acid_4746 | COG5659 FOG: Transposase | none |
| putative transposase | Acid_4751 | pfam04986 Putative transposase | 4 |
| phage integrase family | Acid_4752 | INT_Int1  COG4974 - Site-specific recombinase XerD | 4 |
| transposase IS3/IS911 family | Acid_5157 | COG2963 Transposase and inactivated derivatives  pfam01527 - Transposase_8 | 1 |
| integrase catalytic region | Acid_5158 | pfam00665 rve | 1 |
| integrase catalytic region | Acid_5161 | pfam00665 rve | 2 |
| transposase IS3/IS911 family | Acid_5162 | pfam01527 - Transposase_8 | 2 |
| integrase catalytic region | Acid_5236 | pfam00665 rve  HTH_7 | 6 |
| transposase IS116/IS110/IS902 family | Acid_5320 | pfam01548 Transposase_9 pfam02371 Transposase_20  transposase_IS116/IS110/IS902 | 7 |
| transposase IS116/IS110/IS902 family | Acid_5341 | pfam02371 Transposase_20  transposase_IS116/IS110/IS902 | 11 |
| integrase catalytic region | Acid_5347 | pfam00665 rve  HTH_7 | 6 |
| transposase IS116/IS110/IS902 family | Acid_5398 | pfam01548 Transposase_9 pfam02371 Transposase_20  transposase_IS116/IS110/IS902 | 7 |
| putative transposase protein Y4bF | Acid_5588 | no domains defined | 8 |
| transposase IS116/IS110/IS902 family | Acid_5784 | pfam01548 Transposase_9 pfam02371 Transposase_20  transposase_IS116/IS110/IS902 | 7 |
| transposase IS116/IS110/IS902 family | Acid_5830 | pfam01548 Transposase_9 pfam02371 Transposase_20  transposase_IS116/IS110/IS902 | 7 |
| phage integrase family | Acid_5994 | INT_phiLC3_C  COG4974 - Site-specific recombinase XerD | none |
| transposase IS66 | Acid_6003 | pfam03050 - Transposase_25  COG2251 - Predicted nuclease (RecB family) | none |
| integrase catalytic region | Acid_6059 | pfam00665 rve | 15 |
| integrase catalytic region | Acid_6069 | pfam00665 rve | 2 |
| transposase IS3/IS911 family | Acid_6070 | pfam01527 - Transposase_8 | 2 |
| transposase | Acid_6101 | no domains, fragment? | 5 |
| phage integrase family protein | Acid_6102 | INT_Int1  COG4974 - Site-specific recombinase XerD | 5 |
| putative transposase | Acid_6119 | pfam04986 Putative transposase | none |
| putative transposase | Acid_6124 | no domains | 12 |
| transposase IS116/IS110/IS902 family | Acid_6356 | pfam01548 Transposase_9 pfam02371 Transposase_20  transposase_IS116/IS110/IS902 | 7 |
| transposase IS116/IS110/IS902 family | Acid_6476 | pfam01548 Transposase_9 pfam02371 Transposase_20  transposase_IS116/IS110/IS902 | 7 |
| integrase catalytic region | Acid_6521 | pfam00665 rve | 6 |
| transposase IS116/IS110/IS902 family | Acid_6688 | pfam01548 Transposase_9 pfam02371 Transposase_20  transposase_IS116/IS110/IS902 | 7 |
| transposase | Acid_6781 | no domains, fragment? | none |
| phage integrase family | Acid_6791 | INT_XerDC  COG4974 - Site-specific recombinase XerD | none |
| phage integrase family | Acid_6799 | INT_SG5  COG4974 - Site-specific recombinase XerD | none |
| phage integrase family | Acid_6800 | INT_SG4  COG4974 - Site-specific recombinase XerD | none |
| integrase catalytic region | Acid_6803 | COG3316 | 16 |
| integrase catalytic region | Acid_6807 | pfam00665 rve | none |
| integrase catalytic region | Acid_6822 | pfam00665 rve | 1 |
| transposase IS3/IS911 family | Acid_6823 | pfam01527 - Transposase_8 | 1 |
| transposase | Acid_6843 | no domains | none |
| putative transposase | Acid_6844 | no domains | none |
| transposase IS3/IS911 family | Acid_6861 | pfam01527 - Transposase_8 | 1 |
| integrase catalytic region | Acid_6862 | pfam00665 rve | 1 |
| transposase | Acid_6867 | no domains | none |
| transposase IS204/IS1001/IS1096/IS1165 family | Acid_6898 | COG3464 Transposase and inactivated derivatives  pfam01610 - Transposase_12 | none |
| transposase IS116/IS110/IS902 family | Acid_7041 | pfam01548 Transposase_9 pfam02371 Transposase_20  transposase_IS116/IS110/IS902 | 7 |
| integrase catalytic region | Acid_7104 | pfam00665 rve  HTH_7 | 6 |
| transposase-like | Acid_7107 | KEGG: mxa:MXAN_0516 transposase, IS630 family  pfam01371:Trp repressor protein. This protein binds to tryptophan and represses transcription of the Trp operon. | none |
| integrase catalytic region | Acid_7211 | pfam00665 rve  HTH_7 | 6 |
| transposase IS116/IS110/IS902 family | Acid_7233 | pfam01548 Transposase_9 pfam02371 Transposase_20  transposase_IS116/IS110/IS902 | 7 |
| transposase IS3/IS911 family | Acid_7255 | pfam01527 - Transposase_8 | 3 |
| integrase catalytic region | Acid_7256 | pfam00665 rve | 3 |
| integrase catalytic region | Acid_7608 | pfam00665 rve | 16 |
| integrase catalytic region | Acid_7618 | pfam00665 rve | 15 |
| integrase catalytic region | Acid_7626 | pfam00665 rve | 16 |
| phage integrase family protein | Acid_7640 | DNA-BRE-C | 5 |
| phage integrase family protein | Acid_7641 | DNA-BRE-C, Site-specific recombinase XerD | 10 |
| phage integrase family protein | Acid_7642 | Int_SG4, DNA-BRE-C, site-specific tyrosine recombinase XerC | 14 |
| phage integrase family protein | Acid_7643 | pAE1, DNA-BRE-C, XerC | 9 |
| putative transposase | Acid_7645 | pfam04986 Putative transposase | 5 |
| integrase catalytic region | Acid_7942 | pfam01527 - Transposase_8  pfam00665 rve | 3 |
