# Supplementary material for: Biological Consequences of Ancient Gene Acquisition and Duplication in the Large Genome of Candidatus Solibacter usitatus Ellin6076
Source: PLoS One. 2011 Sep 15;6(9):e24882. doi: 10.1371/journal.pone.0024882 (PMC3174227; doi:10.1371/journal.pone.0024882)
Supplement: Table S3 — Candidate genomic islands in the Ellin6076 genome. (DOC) [file pone.0024882.s010.doc]

**Table S3.** Candidate genomic islands in the Ellin6076 genome

| Region | size/coords | tRNA location | mobile elements/phage/plasmid genes in the region | GC content | dinucleotide bias |
| --- | --- | --- | --- | --- | --- |
| I. Acid_0037 – Acid_0155 | 57495-199752 | Acid_R0002 on + strand between Acid_0139 and Acid_0143 | no  phage Tail Collar domain in  Acid_0149  Acid_0150  Acid_0151 | variable | variable |
|  |  |  |  |  |  |
| II. Acid_0208 – Acid_0379 | 273165-480673 | Acid_R0003 on – strand between Acid_0243 and Acid_0230 | phage integrase family protein Acid_0347 | variable | variable |
|  |  |  |  |  |  |
| III. Acid_0607 – Acid_0665  Good candidate | 745512 - 832975 | Acid_R0007 on + strand between Acid_0604 and Acid_0608 | phage integrase family proteins  Acid_0629  Acid_0630  Acid_0632  Acid_0633  Acid_0635  transposase IS3/IS911 family  Acid_0634  transposase IS204/IS1001/IS1096/IS1165 family Acid_0644  plasmid maintenance system killer  Acid_0688  plasmid maintenance system antidote  Acid_0689  plasmid pRiA4b ORF-3 family protein  Acid_0696 | mostly below average | yes |
|  |  |  |  |  |  |
| IV. Acid_0819 – Acid_1054 | 1017689-1194647 | Acid_R0009 on + strand between Acid_0970 and Acid_0974  Acid_R0011 and Acid_R0012 in an rRNA operon between Acid_1004 and Acid_1007 | integrase catalytic region  Acid_0819  integrase catalytic region  Acid_0942  transposase IS3/IS911 family  Acid_0943  plasmid stabilization system  Acid_1015 | variable | yes |
|  |  |  |  |  |  |
| V.  Acid_1110 – Acid_1160 | 1365753-1431894 | Acid_R0015 on – strand between Acid_1158 and Acid_1161 | integrase catalytic region  Acid_1129 | variable | variable |
|  |  |  |  |  |  |
| VI.  Acid_1216 – Acid-1514 | 1500998-1858185 | Acid_R0016 on – strand between Acid_1369 and Acid-1370  Acid_R0017 on + strand between Acid_1372 and Acid_1373  Acid_R0018 on + strand between Acid_1475 and Acid_1477 | transposase IS116/IS110/IS902 family  Acid_1319  integrase catalytic region  Acid_1329  transposase IS3/IS911 family  Acid_1503  integrase catalytic region  Acid_1504 | variable | variable |
|  |  |  |  |  |  |
| VII.  Acid_1602 – Acid_1770 | 1974941-2200453 | Acid_R0020 and Acid_R0021 on + strand in an rRNA operon between Acid_1601 and Acid-1602  Acid_R0024 on + strand between Acid_1607 and Acid_1609  Acid_R0025 on + strand between Acid_1665 and Acid_1669  Acid_R0026 on + strand between Acid_1770 and Acid_1771 | integrase catalytic region  Acid_1623  Acid_1625  transposase IS3/IS911 family  Acid_1624  Putative bacteriophage-related  Acid_1678  plasmid-related protein  Acid_1609 | variable | variable |
|  |  |  |  |  |  |
| VIII.  Acid_1830 – Acid_2150 | 2282350-2752528 | Acid_R0027 on + strand between Acid_1923 and Acid_1928  Acid_R0028 on – strand between Acid_2113 and Acid_2114 | putative transposase protein, Y4bF  Acid_1944  Acid_1955  integrase catalytic region  Acid_2150 | variable | variable |
|  |  |  |  |  |  |
| IX. Acid_2196 -Acid_3156 | 2814111-3999816 | Acid_R0029 on – strand between Acid_2346 and Acid_2348  Acid_R0030 on – strand between Acid_2688 and Acid_2691  Acid_R0031 on + strand between Acid_2749 and Acid_2752  Acid_R0032 on + strand between Acid_2829 and Acid_2833  Acid_R0033 on + strand between Acid_2895 and Acid_2898 | transposase IS116/IS110/IS902 family  Acid_2198  IS3/IS911 family  Acid_2274  Acid_2460  IS66  Acid_2528  Acid_2529  IS3/IS911 family  Acid_2592  IS116/IS110/IS902 family  Acid_2663  Acid_2712  putative transposase  Acid_2778  IS66  Acid_2940  Acid_2942  Acid_2946  IS3/IS911 family  Acid_3155  Integrase catalytic region  Acid_2230  Acid_2273  Acid_2459  phage integrase family  Acid_2520  Acid_2521  Acid_2522  integrase catalytic region  Acid_2593  phage integrase family  Acid_2776  Acid_2943  Acid_2945  integrase catalytic region  Acid_3156  plasmid maintenance system antidote protein  Acid_2330  plasmid maintenance system killer  Acid_2331  phage SPO1 DNA polymerase-related protein  Acid_2350  phage shock protein A  Acid_2384 | variable | variable |
|  |  |  |  |  |  |
| X. Acid_3253 – Acid_4625  a lot of mobile elements, tRNA genes and small areas of dinucleotide bias – may have been an island at one time, but subsequent rearrangements obscured the boundaries? | 4117534-5850429 |  | phage shock protein C  Acid_4337  phage shock protein A  Acid_4570 | variable | variable |
|  |  |  |  |  |  |
| XI. Acid_4625 – Acid_4695  Good candidate | 5850429-5944933 | Acid_R0043 on – strand between Acid_4694 and Acid_4696 | Integrase catalytic region  Acid_4625 | mostly below average | yes |
|  |  |  |  |  |  |
| XII. Acid_4696 – Acid_5994  a lot of mobile elements, tRNA genes and small areas of dinucleotide bias – may have been an island at one time, but subsequent rearrangements obscured the boundaries? | 5945173-7519982 |  | bacteriophage N4 receptor  Acid_5606 | variable | variable |
|  |  |  |  |  |  |
| XIII.  Acid_5980 – Acid_6176  Good candidate | 7502911-7745505 | Acid_R0050 on + strand between Acid_5992 and Acid_5997  Acid_R0051 on + strand between Acid_6067 and Acid_6072 | Transposase IS66  Acid_6003  transposase  Acid_6101  putative transposase  Acid_6119  IS66  Acid_6121  putative transposase  Acid_6124  Integrase catalytic region  Acid_6059  Acid_6069  phage integrase family  Acid_6102 | variable | variable |
|  |  |  |  |  |  |
| XIV.  Acid_6243 – Acid_6638  hard to find any boundaries here | 7825707-8326556 |  | phage Gp37Gp68 family protein  Acid_6594  plamid stabilization system  Acid_6411 | variable | variable |
|  |  |  |  |  |  |
| XV. Acid_6758 – Acid_6949  Good candidate | 8469801-8733305 | Acid_R0057 on + strand between Acid_6765 and Acid_6770 | transposase  Acid_6781  IS3/IS911  Acid_6823  transposase  Acid_6843  putative transposase  Acid_6844  IS3/IS911  Acid_6861  transposase  Acid_6867  IS204/IS1001/IS1096/IS1165 family  Acid_6898  phage integrase family  Acid_6791  Acid_6799  Acid_6800  integrase catalytic region  Acid_6803  Acid_6807  Acid_6822  Acid_6862 | mostly below average | yes |
|  |  |  |  |  |  |
| XVI.  Acid_7211 – Acid_7657 | 9059709-9616304 | Acid_R0058 on – strand between Acid_7657 and Acid_7658 | integrase catalytic region  Acid_7211  transposase IS116/IS110/IS902 family  Acid_7233  transposase IS3/IS911 family  Acid_7255  integrase catalytic region  Acid_7256  Acid_7608  Acid_7618  Acid_7626  phage integrase family protein  Acid_7640  Acid_7641  Acid_7642  Acid_7643  putative transposase  Acid_7645  plasmid-encoded RepA protein  Acid_7603  plasmid-related protein  Acid_7627 | variable | variable |
