# Supplementary material for: Biological Consequences of Ancient Gene Acquisition and Duplication in the Large Genome of Candidatus Solibacter usitatus Ellin6076
Source: PLoS One. 2011 Sep 15;6(9):e24882. doi: 10.1371/journal.pone.0024882 (PMC3174227; doi:10.1371/journal.pone.0024882)
Supplement: Table S4 — Distribution of genes in COG categories for Acidobacteria strains Ellin6076 and Ellin345, compared to other large-small genome pairs. (DOC) [file pone.0024882.s011.doc]

**Table S4**. Distribution of genes in COG categories for *Acidobacteria* strains Ellin6076 and Ellin345, compared to other large-small genome pairs.

| **COG Category** | 6076 | 345 | MLOT | BNC1 | MC2 155 | MLEP | HCHE | MED92 | REU | RSO |
| --- | --- | --- | --- | --- | --- | --- | --- | --- | --- | --- |
| **Information storage and processing** |  |  |  |  |  |  |  |  |  |  |
| A. RNA processing and modification | 0 | 1 | 0 | 0 | 3 | 1 | 2 | 1 | 1 | 5 |
| B. Chromatin structure and dynamics | 3 | 2 | 5 | 1 | 1 | 0 | 3 | 1 | 3 | 2 |
| J. Translation, ribosomal structure and biogenesis | 183 | 164 | 198 | 169 | 177 | 123 | 191 | 182 | 207 | 155 |
| K. Transcription | 471 | 295 | 569 | 289 | 525 | 74 | 397 | 251 | 604 | 314 |
| L. Replication, recombination and repair | 328 | 158 | 294 | 164 | 193 | 65 | 212 | 126 | 192 | 212 |
| **Cellular processes and signaling** |  |  |  |  |  |  |  |  |  |  |
| D. Cell cycle, cell division, chromosome partitioning | 26 | 25 | 34 | 35 | 28 | 21 | 40 | 32 | 37 | 29 |
| M. Cell wall/membrane/envelope biogenesis | 443 | 257 | 313 | 228 | 170 | 69 | 269 | 168 | 301 | 242 |
| N. Cell motility | 101 | 82 | 67 | 51 | 6 | 6 | 185 | 121 | 113 | 129 |
| O. Posttranslational modification, protein turnover, chaperones | 183 | 134 | 205 | 163 | 136 | 62 | 201 | 143 | 219 | 151 |
| T. Signal transduction mechanisms | 421 | 316 | 254 | 135 | 190 | 39 | 474 | 380 | 302 | 212 |
| U. Intracellular trafficking, secretion and vesicular transport | 172 | 108 | 141 | 140 | 24 | 20 | 142 | 84 | 144 | 167 |
| V. Defense mechanisms | 236 | 116 | 67 | 40 | 49 | 12 | 69 | 34 | 65 | 46 |
| W. Extracellular structures | 0 | 0 | 1 | 0 | 0 | 0 | 0 | 0 | 0 | 1 |
| Y. Nuclear structure | 0 | 0 | 0 | 0 | 0 | 0 | 0 | 0 | 0 | 0 |
| Z. Cytoskeleton | 0 | 0 | 0 | 0 | 0 | 0 | 1 | 0 | 0 | 0 |
| **Metabolism** |  |  |  |  |  |  |  |  |  |  |
| C. Energy production and conversion | 344 | 223 | 328 | 268 | 504 | 78 | 215 | 214 | 506 | 235 |
| E. Amino acid transport and metabolism | 392 | 268 | 740 | 508 | 485 | 129 | 421 | 304 | 520 | 364 |
| F. Nucleotide transport and metabolism | 93 | 68 | 91 | 86 | 103 | 55 | 88 | 67 | 97 | 72 |
| G. Carbohydrate transport and metabolism | 427 | 213 | 524 | 247 | 416 | 72 | 234 | 101 | 275 | 188 |
| H. Coenzyme transport and metabolism | 191 | 131 | 233 | 166 | 205 | 87 | 191 | 156 | 197 | 168 |
| I. Lipid transport and metabolism | 182 | 111 | 269 | 168 | 498 | 91 | 169 | 114 | 415 | 169 |
| P. Inorganic ion transport and metabolism | 179 | 118 | 286 | 239 | 282 | 49 | 205 | 143 | 303 | 186 |
| Q. Secondary metabolites biosynthesis, transport and catabolism | 148 | 70 | 202 | 123 | 403 | 57 | 130 | 63 | 258 | 100 |
| **Poorly characterized** |  |  |  |  |  |  |  |  |  |  |
| R. General function prediction only | 833 | 473 | 772 | 462 | 821 | 127 | 544 | 334 | 697 | 418 |
| S. Function unknown | 477 | 308 | 643 | 420 | 347 | 81 | 418 | 278 | 617 | 348 |

SUS: “*Solibacter usitatus”*, strain Ellin 6076

345: *“Koreobacter versatilis”*, strain Ellin 345

MLOT: *Mesorhizobium loti* MAFF303099

BNC1: *Mesorhizobium* sp. BNC1

MC2 155: *Mycobacterium smegmatis* MC2 155

MLEP: *Mycobacterium leprae* TN

HCHE: *Hahella chejuensis* KCTC 2396

MED92: *Oceanospirillum* sp. MED92

REU: *Ralstonia eutropha* H16

RSO: *Ralstonia solanacearum* UW551

NOTE: Some genes are present in more than one COG category
