# Supplementary material for: Biological Consequences of Ancient Gene Acquisition and Duplication in the Large Genome of Candidatus Solibacter usitatus Ellin6076
Source: PLoS One. 2011 Sep 15;6(9):e24882. doi: 10.1371/journal.pone.0024882 (PMC3174227; doi:10.1371/journal.pone.0024882)
Supplement: Table S5 — Distribution of genes in COG categories for Acidobacteria strains Ellin6076 and Ellin345. (DOC) [file pone.0024882.s012.doc]

**Table S5.** Distribution of genes in COG categories for *Acidobacteria* strains Ellin6076 and Ellin345. Only categories with pronounced differences are shown. Shaded categories depict at least a two-fold increase in gene content. The complete list of COG categories and comparisons can be obtained through the Integrated Microbial Genomes system (http://img.jgi.doe.gov/cgi-bin/pub/main.cgi).

| COG CATEGORY | Strain Ellin6076 | Strain Ellin345 | Fold Increase+ |
| --- | --- | --- | --- |
| **Information storage and processing** |  |  |  |
| (A) RNA processing and modification | 0 | 1 | 0 |
| (B) Chromatin structure and dynamics | 3 | 2 | 1.5 |
| (J) Translation | 183 | 164 | 1.1 |
| Asp-tRNAAsn/Glu-tRNAGln amidotransferase A subunit and related | 13 | 6 | 2.2 |
| (K) Transcription | 471 | 295 | 1.6 |
| Serine/threonine protein kinase | 79 | 30 | 2.6 |
| DNA-directed RNA polymerase, sigma 24 homolog | 70 | 28 | 2.5 |
| Transcriptional regulators | 114 | 53 | 2.2 |
| Response regulators | 141 | 119 | 1.2 |
| (L) Replication, recombination, repair | 328 | 158 | 2.1 |
| Serine/threonine protein kinase | 79 | 30 | 2.6 |
| Site-specific recombinase XerD | 32 | 6 | 5.3 |
| Transposase and inactivated derivatives | 64 | 10 | 6.4 |
| **Cellular processes** |  |  |  |
| (D) Cell cycle control, mitosis, meiosis | 26 | 25 | 1.0 |
| (M) Cell wall/membrane biogenesis | 444 | 257 | 1.7 |
| Glycosyltransferase | 36 | 15 | 2.4 |
| 4-amino-4-deoxy-L-arabinose transferase and related | 26 | 7 | 3.7 |
| Glycosyltransferases involved in cell wall biogenesis | 36 | 15 | 2.4 |
| L-alanine-DL-glutamate epimerase and related | 25 | 4 | 6.3 |
| Outer membrane protein | 40 | 17 | 2.4 |
| Nucleoside-diphosphate-sugar epimerases | 29 | 20 | 1.5 |
| ABC-type transport system, involved in lipoprotein release, permease | 17 | 11 | 1.6 |
| Membrane-fusion protein | 14 | 8 | 1.8 |
| Periplasmic protease | 13 | 2 | 6.5 |
| Endopolygalacturonase | 11 | 0 | > 11.0 |
| Dihydropicolinate synthase/N-acetylneuraminate lyase | 9 | 2 | 4.5 |
| Cell division protein FtsI/penicillin-binding protein 2 | 7 | 4 | 1.8 |
| Outer membrane protein/protective antigen OMA87 | 6 | 3 | 2.0 |
| ABC-type polysaccharide/polyol phosphate export system, permease | 6 | 0 | > 6.0 |
| ABC-type polysaccharide/polyol phosphate export system, ATPase | 6 | 0 | > 6.0 |
| Periplasmic protein TonB | 6 | 11 | NA |
| Soluble lytic murein transglycolase and related regulatory proteins | 6 | 4 | 1.5 |
| ADP-heptose:LPS heptosyltransferase | 5 | 2 | 2.5 |
| Membrane proteins related to metalloendopeptidases | 5 | 1 | 5.0 |
| Small-conductance mechanosensitive channel | 5 | 2 | 2.5 |
| Sortase | 6 | 1 | 6.0 |
| Lipid A core-O-antigen ligase and related | 4 | 2 | 2.0 |
| (N) Cell motility | 101 | 84 | 1.2 |
| Tfp pilus assembly protein PilF | 26 | 10 | 2.6 |
| Type II secretory pathway, pseudopilin PulG | 5 | 9 | NA |
| Type II secretory pathway, component PulD | 4 | 2 | 2.0 |
| Methyl-accepting chemotaxis protein | 4 | 3 | 1.3 |
| Flp pilus assembly protein TadC | 3 | 0 | > 3.0 |
| Predicted periplasmic or secreted lipoprotein | 3 | 0 | > 3.0 |
| (O) Posttranslational modification, protein turnover, chaperones | 183 | 134 | 1.4 |
| Perirredoxin | 21 | 9 | 2.3 |
| Protein disulfide isomerase | 8 | 4 | 2.0 |
| ATPases with chaperone activity, ATP-binding subunit | 8 | 3 | 2.7 |
| Trypsin-like serine proteases, typically periplasmic, C-terminal PDZ | 8 | 4 | 2.0 |
| Peptidyl-prolyl cis-trans isomerase (rotamase) – cyclophilin family | 7 | 2 | 3.5 |
| Molecular chaperone | 10 | 5 | 2.0 |
| (T) Signal transduction mechanisms | 421 | 316 | 1.3 |
| Serine/threonine protein kinase | 79 | 30 | 2.6 |
| Response regulators | 141 | 119 | 1.2 |
| Signal transduction histidine kinase | 88 | 58 | 1.5 |
| Antirepressor regulating drug resistance, signal transduction comp. | 12 | 3 | 4.0 |
| Bacteriophytochrome | 9 | 2 | 4.5 |
| DnaK suppressor protein | 7 | 2 | 3.5 |
| (U) Intracellular trafficking, secretion | 174 | 110 | 1.6 |
| Tfp pilus assembly protein PilF | 26 | 10 | 2.6 |
| Outer membrane protein | 25 | 10 | 2.5 |
| Periplasmic component of Tol biopolymer transport system | 18 | 7 | 2.6 |
| Flp pilus assembly protein TadD | 17 | 9 | 1.9 |
| Flp pilus assembly protein, ATPase CpaE | 4 | 0 | > 4.0 |
| Flp pilus assembly protein, ATPase CpaF | 4 | 0 | > 4.0 |
| Type II secretory pathway, component PulD | 4 | 2 | 2.0 |
| Signal peptidase I | 4 | 3 | 1.3 |
| Flp pilus assembly protein TadB | 3 | 0 | > 3.0 |
| Flp pilus assembly protein CpaB | 3 | 0 | > 3.0 |
| Flp pilus assembly protein TadC | 3 | 0 | > 3.0 |
| Flp pilus assembly protein, secretin CpaC | 2 | 0 | > 2.0 |
| Flp pilus assembly protein TadG | 2 | 0 | > 2.0 |
| Flp pilus assembly protein, pilin Flp | 2 | 0 | > 2.0 |
| Flp pilus assembly protein, protease CpaA | 1 | 0 | > 1.0 |
| (V) Defense mechanisms | 236 | 116 | 2.0 |
| ABC-type antimicrobial peptide transport system | 124 | 54 | 2.6 |
| ABC-type multidrug transport system | 44 | 21 | 2.1 |
| Beta-lactamase class C and other penicillin binding proteins | 23 | 13 | 1.8 |
| Multidrug resistance efflux pump | 18 | 9 | 2.0 |
| Cation/multidrug efflux pump | 19 | 8 | 2.4 |
| Lantibiotic modifying enzyme | 2 | 0 | > 2.0 |
| (W) Extracellular structures | 0 | 0 | 0 |
| (Z) Cytoskeleton | 0 | 0 | 0 |
| **Metabolism** |  |  |  |
| (C) Energy production, conversion | 344 | 223 | 1.5 |
| Predicted oxidoreductases | 20 | 8 | 2.5 |
| Aerobic-type carbon monoxide dehydrogenase, small subunit | 9 | 3 | 3.0 |
| Aerobic-type carbon monoxide dehydrogenase, large subunit | 8 | 3 | 2.7 |
| Glycosyltransferases, related to UDP-glucuronosyltransferase | 8 | 3 | 2.7 |
| FAD/FMN-containing dehydrogenases | 8 | 2 | 4.0 |
| Carbon dioxide conc. mechanism/carboxysome shell proteins | 9 | 0 | > 9.0 |
| FOG:HEAT repeat | 6 | 0 | > 6.0 |
| Rieske Fe-S protein | 5 | 0 | > 5.0 |
| Predicted acetamidase/formamidase | 4 | 0 | > 4.0 |
| Cytochrome b subunit | 4 | 0 | > 4.0 |
| Cytochrome c2 | 3 | 0 | > 3.0 |
| (E) Amino acid transport, metabolism | 392 | 268 | 1.5 |
| Dipeptidyl aminopeptidases/acylaminoacylpeptidases | 25 | 10 | 2.5 |
| Acetylornithine deacetylase and related | 17 | 5 | 3.4 |
| Lysophospholipase L1 and related esterases | 16 | 1 | 16.0 |
| Threonine dehydrogenase and related Zn-dependent dehydrogenases | 12 | 5 | 2.4 |
| Aspartate/tyrosine/aromatic aminotransferase | 9 | 6 | 1.5 |
| Dihydropicolinate synthase/N-acetylneuraminate lyase | 9 | 2 | 4.5 |
| Choliine dehydrogenase and related | 8 | 2 | 4.0 |
| Xaa-Pro aminopeptidase | 7 | 3 | 3.5 |
| Histidinol phosphate/aromatic aminotransferase | 6 | 3 | 2.0 |
| Thiamine pyrophosphate-requiring enzymes | 6 | 3 | 2.0 |
| Threonine dehydratase | 5 | 2 | 2.5 |
| Spermidine synthase | 5 | 0 | > 5.0 |
| Asparagine synthase (glutamate hydrolyzing) | 4 | 0 | > 4.0 |
| ABC-type spremidine/putrescine transport systems, ATPase | 3 | 1 | 3.0 |
| Predicted ornithine cyclodeaminase | 3 | 0 | > 3.0 |
| Methionine synthase II (cobalamin-independent) | 3 | 0 | > 3.0 |
| (F) Nucleotide transport, metabolism | 93 | 68 | 1.4 |
| ADP-ribose pyrophosphatase | 6 | 2 | 3.0 |
| Inosine-uridine nucleoside N-ribohydrolase | 3 | 0 | > 3.0 |
| 5’-nucleotidase/2’,3’-cyclic phosphodiesterase and related esterases | 2 | 0 | > 2.0 |
| (G) Carbohydrate transport, metabolism | 427 | 213 | 2.0 |
| Sugar phosphate isomerases/epimerases | 41 | 6 | 6.8 |
| Arabinose efflux permease | 27 | 12 | 2.3 |
| Glucose dehydrogenase | 26 | 0 | > 26.0 |
| Nucleoside-diphosphate-sugar epimerases | 29 | 20 | 1.5 |
| Sugar phosphate permease | 16 | 6 | 2.7 |
| Gluconolactonase | 13 | 2 | 6.5 |
| Sugar kinases, ribokinase family | 10 | 3 | 3.3 |
| Beta-galactosidase/beta-glucuronidase | 9 | 4 | 2.3 |
| Alpha-L-arabinofuranosidase | 8 | 2 | 4.0 |
| Glycosyltransferases, related to UDP-glucuronosyltransferase | 8 | 3 | 2.7 |
| Alpha-L-fucosidase | 7 | 1 | 7.0 |
| Glucose/sorbosone dehydrogenases | 6 | 1 | 6.0 |
| ABC-type polysaccharide/polyol phosphate export system, permease | 6 | 0 | > 6.0 |
| ABC-type polysaccharide/polyol phosphate export system, ATPase | 6 | 0 | > 6.0 |
| Fucose permease | 6 | 3 | 2.0 |
| Predicted xylanase/chitin deacetylase | 6 | 5 | 1.2 |
| 2,4-dihydroxyhept-2-ene-1,7-dioic acid aldolase | 5 | 0 | > 5.0 |
| Beta-glucosidase and related glycosidases | 5 | 8 | NA |
| Mannose-6-phosphate isomerase | 5 | 3 | 1.7 |
| Ribulose-5-phosphate 4-epimerase and related | 5 | 3 | 1.7 |
| Beta-xylosidase | 4 | 0 | > 4.0 |
| Beta-galactosidase | 4 | 1 | 4.0 |
| Beta-1,4-xylanase | 3 | 0 | > 3.0 |
| Hydroxypyruvate isomerase | 3 | 0 | > 3.0 |
| D-mannonate dehydratase | 3 | 0 | > 3.0 |
| Ribose/xylose/arabinose/galactoside ABC-type transport, permease | 3 | 0 | > 3.0 |
| (H) Coenzyme transport, metabolism | 191 | 131 | 1.5 |
| Methylase involved in ubiquinone/menaquinone biosynthesis | 37 | 19 | 2.0 |
| 2-polyprenyl-3-methyl-5-hydroxy-6-metoxy-1,4-benzoquinol methylase | 18 | 4 | 4.5 |
| Mg-chelatase subunit ChlD | 8 | 4 | 2.0 |
| Demethylmenaquinone methyltransferase | 7 | 0 | > 7.0 |
| (I) Lipid transport, metabolism | 182 | 110 | 1.7 |
| Dehydrogenases with different specificities | 39 | 15 | 2.6 |
| Esterase/lipase | 11 | 5 | 2.2 |
| Carboxylesterase type B | 9 | 0 | > 9.0 |
| Acyl-CoA synthetases (AMP-forming) | 8 | 4 | 2.0 |
| (P) Inorganic ion transport, metabolism | 179 | 118 | 1.5 |
| Arylsulfatase A and related enzymes | 18 | 0 | > 18.0 |
| Enterochelin esterase and related enzymes | 16 | 2 | 8.0 |
| Outer membrane receptor for ferrienterochelin and colicins | 7 | 3 | 2.3 |
| Mn2+ and Fe2+ transporters of the NRAMP family | 6 | 3 | 3.0 |
| Cytochrome c peroxidase | 6 | 0 | > 6.0 |
| Putative silver efflux pump | 3 | 1 | 3.0 |
| (Q) Secondary metabolites | 148 | 70 | 2.1 |
| Dehydrogenases with different specificities | 39 | 15 | 2.6 |
| Imidazoloneproprionase and related amidohydrolases | 21 | 11 | 1.9 |
| Dienelactone hydrolase and related enzymes | 8 | 1 | 8.0 |
| Acyl-CoA synthetases (AMP-forming) | 8 | 4 | 2.0 |
| Carbon dioxide conc. mechanism/carboxysome shell proteins | 9 | 0 | > 9.0 |
| N-acyl-D-aspartate/D-glutamate deacylase | 5 | 2 | 2.5 |
| Acyl carrier protein | 5 | 3 | 1.7 |
| Protein involved in biosynthesis of mitomycin antibiotics/fumonisin | 4 | 0 | > 4.0 |
| Predicted enzyme involved in methoxymalonyl-ACP biodynthesis | 4 | 1 | 4.0 |
| 3-oxoacyl-(acyl-carrier-protein) synthase | 4 | 2 | 2.0 |
| Polyketide synthase modules and related | 3 | 1 | 3.0 |
| **Poorly characterized** |  |  |  |
| (R) General function prediction | 833 | 472 | 1.8 |
| (S) Function unknown | 477 | 308 | 1.5 |
| (*) Not assigned | 2943 | 1612 | 1.8 |

+ Not normalized for genome size
