# Supplementary material for: Biological Consequences of Ancient Gene Acquisition and Duplication in the Large Genome of Candidatus Solibacter usitatus Ellin6076
Source: PLoS One. 2011 Sep 15;6(9):e24882. doi: 10.1371/journal.pone.0024882 (PMC3174227; doi:10.1371/journal.pone.0024882)
Supplement: Table S6 — Average genome size of representative Acidobacteria isolates from subdivisions 1 and 3, determined by pulse field gel electrophoresis. (DOC) [file pone.0024882.s013.doc]

**Table S6.** Average genome size of representative *Acidobacteria* isolates from subdivisions 1 and 3, determined by pulse field gel electrophoresis.

| Strain | Mean genomic size ± std. dev. (Kb) (restriction enzyme used, number of independent determinations) | Strain reference |
| --- | --- | --- |
| **Subdivision 1** |  |  |
| *A. capsulatum* | 4,128 | [22] |
| Ellin345 | 5,650 | [22] |
| Strain TAA 166 | 4,036 ± 325 (*SwaI*, n=2)  3,817 ± 128 (*PmeI*, n=2) | This study |
| *T. roseus*, strain KBS 63 | 2,535 ± 0 (*SwaI*, n=1)  2,292 ± 352 (*PmeI*, n=2) | This study |
| *T.* sp., strain TAA 43 | 2,069 ± 206 (*SwaI*, n=3)  2,457 ± 221 (*PmeI*, n=7) | This study |
|  |  |  |
| **Subdivision 3** |  |  |
| Ellin6076 | 9,967 | [22] |
| *B. aggregatus,* strain MPL3 | 5,869 ± 59 (*SwaI*, n=4)  5,829 ± 58 (*PmeI*, n=5) | This study |
