# Supplementary material for: Biological Consequences of Ancient Gene Acquisition and Duplication in the Large Genome of Candidatus Solibacter usitatus Ellin6076
Source: PLoS One. 2011 Sep 15;6(9):e24882. doi: 10.1371/journal.pone.0024882 (PMC3174227; doi:10.1371/journal.pone.0024882)
Supplement: Table S7 — Expansion of COG categories for carbohydrate transport and metabolism. (DOC) [file pone.0024882.s014.doc]

**Table S7**. Expansion of COG categories for carbohydrate transport and metabolism.

| Carbon source | genes | # copies in Ellin345 | # copies in Ellin6076 |
| --- | --- | --- | --- |
| total all sources | all genes in carbohydrate metabolism COG category | 213 | 427 |
| Specific functions* |  |  |  |
| plant polymers |  |  |  |
| cellulose | cellulase M (EC:3.2.1.4) | 0 | 1 |
|  | beta-glucosidase  (EC:3.2.1.21) | 9 | 6 |
|  | endoglucanase (glycosyl hydrolase family 5) | 1 | 1 |
| xylan | beta-xylosidase (EC:3.2.1.99)/ Alpha-N-arabinofuranosidase ( EC:3.2.1.55 ) | 0 | 4 |
|  | xylan 1,4-beta-xylosidase (EC:3.2.1.37) | 1 | 2 |
|  | alpha-L-arabinofuranosidase (EC:3.2.1.55) | 2 | 8 |
|  | alpha-glucuronidase (EC:3.2.1.139) | 1 | 2 |
|  | endo-1,4-beta-xylanase ( EC:3.2.1.8 ) | 0 | 3 |
|  | putative xylanase/chitin deacetylase | 5 | 6 |
| pectin | pectinesterase ( EC:3.1.1.11 ) | 0 | 2 |
|  | polygalacturonase (EC:3.2.1.15) | 0 | 11 |
| chitin | putative xylanase/chitin deacetylase | 5 | 6 |
|  | chitinase | 0 | 2 |
| starch | alpha-amylase/alpha-mannosidase | 2 | 1 |
|  | glucoamylase | 2 | 2 |
| simple sugars |  |  |  |
| arabinose | L-fucose isomerase [Alternative L-arabinose isomerase (EC 5.3.1.4)] | 0 | 1 |
|  | L-arabinose isomerase [EC:5.3.1.4] | 1 | 0 |
|  | L-ribulose 5-phosphate 4-epimerase (EC 5.1.3.4) | 3 | 5 |
| cellobiose | cellobiose phosphorylase | 0 | 1 |
| fructose | pyrophosphate-dependent phosphofructokinase (EC 2.7.1.90) | 3 | 1 |
| galactose | putative aldose 1-epimerase | 0 | 1 |
|  | aldose 1-epimerase (EC 5.1.3.3) | 2 | 3 |
|  | galactokinase | 1 | 2 |
| galacturonate | uronate isomerase [EC:5.3.1.12] | 1 | 1 |
| glucuronate | uronate isomerase [EC:5.3.1.12] | 1 | 1 |
| glucose | Glucose/sorbosone dehydrogenase | 1 | 6 |
|  | glucose dehydrogenase ( EC:1.1.5.2 ) | 0 | 26 |
|  | gluconolactonase | 2 | 13 |
| mannose | mannose-6-phosphate isomerase | 3 | 7 |
| ribose | ribokinase | 3 | 10 |
| xylose | xylose isomerase | 6 | 41 |
| sugar transporters | sugar phosphate permease | 6 | 16 |
|  | arabinose efflux permease | 12 | 27 |
|  | Na+/melibiose and related symporter | 1 | 2 |
|  | major facilitator superfamily | 18 | 40 |
|  | ABC-type polysaccharide/polyol phosphate transport system, | 0 | 6 ATPase component  6 permease component |
| glycoside hydrolases (all families) |  | 16 | 33 |
| sugar kinases | ribokinase family | 3 | 10 |
|  | transcriptional reg./sugar kinase | 6 | 7 |
|  | pentulose and hexulose kinases | 1 | 2 |
|  |  |  |  |

*Only those with pronounced differences between Ellin6076 and Ellin345 are shown.
