# Supplementary material for: Biological Consequences of Ancient Gene Acquisition and Duplication in the Large Genome of Candidatus Solibacter usitatus Ellin6076
Source: PLoS One. 2011 Sep 15;6(9):e24882. doi: 10.1371/journal.pone.0024882 (PMC3174227; doi:10.1371/journal.pone.0024882)
Supplement: Table S8 — Expansion of COG categories for amino acid transport and metabolism. (DOC) [file pone.0024882.s015.doc]

**Table S8**. Expansion of COG categories for amino acid transport and metabolism.

| Function | genes | # copies in Ellin345 | # copies in Ellin6076 |
| --- | --- | --- | --- |
| Total all functions | all genes in amino acid metabolism COG category | 268 | 392 |
| Specific functions* |  |  |  |
| Dipeptidyl aminopeptidases/acylaminoacyl-peptidases |  | 10 | 25 |
|  | peptidase S9, prolyl oligopeptidase (EC:3.4.19.) | 7 | 15 |
|  | peptidase S9B, dipeptidylpeptidase IV domain protein | 0 | 1 |
|  | dipeptidyl-peptidase IV (EC:3.4.14.5). Serine peptidase. MEROPS family S09B | 1 | 1 |
|  | glutamyl peptidase. Serine peptidase. MEROPS family S09D | 0 | 2 |
|  | General function only | 2 | 6 |
| Amino acid transporters |  | 17 | 18 |
|  | amino acid/polyamine/organocation transporter, APC superfamily (TC 2.A.3) | 14 | 13 |
|  | ethanolamine:proton symporter, EAT family (TC 2.A.3.5.1) | 0 | 1 |
|  | arginine:ornithine antiporter, APA family (TC 2.A.3.2.3) | 0 | 1 |
|  | putrescine:proton symporter, AAT family (TC 2.A.3.1.13 | 1 | 0 |
|  | General function only | 2 | 3 |
| Acetylornithine deacetylase/Succinyl-diaminopimelate desuccinylase and related deacylases |  | 5 | 17 |
|  | peptidase M20 ( EC:3.4.17.11 ) | 4 | 10 |
|  | peptidase dimerisation domain protein ( EC:3.5.1.16 /EC:3.4.17.11 ) | 1 | 3 |
|  | amidase, hydantoinase/carbamoylase family ( EC:3.5.1.87 ) | 0 | 3 |
|  | acetylornithine deacetylase (EC 3.5.1.16) | 0 | 1 |
| Lysophospholipase L1 and related esterases |  | 1 | 16 |
|  | lipolytic enzyme, G-D-S-L | 1 | 14 |
|  | Pectate lyase | 0 | 1 |
|  | putative O-antigen related protein | 0 | 1 |
|  |  |  |  |
| Threonine dehydrogenase and related Zn-dependent dehydrogenases |  | 5 | 12 |
|  | Alcohol dehydrogenase GroES-like/Zn-binding ( EC:1.1.1.14 ) |  |  |
|  | L-threonine 3-dehydrogenase (EC 1.1.1.103) |  |  |
|  | oxidoreductase domain protein | 0 | 1 |
| Dihydrodipicolinate synthase/N-acetylneuraminate lyase |  | 2 | 9 |
|  | dihydrodipicolinate synthase (EC 4.2.1.52) | 2 | 9 |
| Aspartate/tyrosine/aromatic aminotransferase |  | 6 | 9 |
|  | aminotransferase, class I and II ( EC:2.6.1.- ) | 5 | 8 |
|  | L-aspartate aminotransferase apoenzyme (EC 2.6.1.1) | 1 | 1 |
| Choline dehydrogenase and related flavoproteins |  |  |  |
|  | glucose-methanol-choline oxidoreductase ( EC:1.1.3.6 ) | 2 | 6 |
|  | Quinoprotein glucose dehydrogenase ( EC:1.1.5.2 ) | 0 | 1 |
|  | 4Fe-4S ferredoxin, iron-sulfur binding domain protein ( EC:1.1.3.6 ) | 0 | 1 |
| Xaa-Pro aminopeptidase |  | 3 | 7 |
|  | peptidase M24 ( EC:3.4.11.9 ) | 2 | 3 |
|  | aminopeptidase P (EC:3.4.11.9) | 1 | 3 |
|  | putative metal-dependent dipeptidase | 0 | 1 |
| Thiamine pyrophosphate-requiring enzymes [acetolactate synthase, pyruvate dehydrogenase (cytochrome), glyoxylate carboligase, phosphonopyruvate decarboxylase] |  | 3 | 6 |
|  | pyruvate oxidase (EC 1.2.3.3) | 1 | 1 |
|  | thiamine pyrophosphate enzyme-like TPP bindin ( EC:2.2.1.6 ) | 1 | 3 |
|  | acetolactate synthase, large subunit, biosynthetic type ( EC:2.2.1.6 ) | 1 | 2 |
| Histidinol-phosphate/aromatic aminotransferase and cobyric acid decarboxylase |  | 3 | 6 |
|  | histidinol-phosphate aminotransferase ( EC:2.6.1.9 ) | 3 | 6 |
| histidinol-phosphate aminotransferase ( EC:2.6.1.9 ) |  | 2 | 5 |
|  | D-isomer specific 2-hydroxyacid dehydrogenase, NAD-binding | 1 | 3 |
|  | D-3-phosphoglycerate dehydrogenase (EC 1.1.1.95) | 1 | 2 |
| Lactoylglutathione lyase and related lyases |  | 4 | 5 |
|  | Glyoxalase/bleomycin resistance protein/dioxygenase ( EC:4.4.1.5 ) | 4 | 5 |
| Na+/proline symporter |  | 4 | 5 |
|  |  |  |  |
| Threonine dehydratase |  | 2 | 5 |
|  | L-threonine ammonia-lyase (EC 4.3.1.19) | 2 | 5 |
|  |  |  |  |
| Only in Ellin6076 |  |  |  |
|  | Spermidine synthase | 0 | 5 |
|  | Asparagine synthase (glutamine-hydrolyzing) | 0 | 4 |
|  | Methionine synthase II (cobalamin-independent) | 0 | 3 |
|  | Predicted ornithine cyclodeaminase, mu-crystallin homolog | 0 | 3 |
|  | Zn-dependent oligopeptidases | 0 | 2 |
|  | Alanine dehydrogenase | 0 | 2 |
|  | ABC-type amino acid transport/signal transduction systems, periplasmic component/domain | 0 | 2 |
|  | Histidinol phosphatase and related hydrolases of the PHP family | 0 | 2 |
|  | Allophanate hydrolase subunit 2 | 0 | 2 |
|  | Allophanate hydrolase subunit 1 | 0 | 2 |
|  | Glutaminase | 0 | 2 |
|  | L-asparaginase/archaeal Glu-tRNAGln amidotransferase subunit D | 0 | 1 |
|  | ABC-type branched-chain amino acid transport systems, periplasmic component | 0 | 1 |
|  | Glutathionylspermidine synthase | 0 | 1 |
|  | Na+/glutamate symporter | 0 | 1 |
|  | ABC-type dipeptide/oligopeptide/nickel transport system, ATPase component | 0 | 1 |
|  | Arginine decarboxylase (spermidine biosynthesis) | 0 | 1 |
|  | ABC-type proline/glycine betaine transport systems, permease component | 0 | 1 |
|  | N-acetylglutamate synthase (N-acetylornithine aminotransferase) | 0 | 1 |
|  | Arginine deiminase | 0 | 1 |
|  | H+/gluconate symporter and related permeases | 0 | 1 |
|  | H+/gluconate symporter and related permeases | 0 | 1 |
|  | Dipeptide/tripeptide permease | 0 | 1 |
|  | 3-deoxy-D-arabino-heptulosonate 7-phosphate (DAHP) synthase | 0 | 1 |
|  | 3-deoxy-D-arabino-heptulosonate 7-phosphate (DAHP) synthase | 0 | 1 |
|  | Acetolactate synthase | 0 | 1 |
|  | Ethanolamine ammonia-lyase, small subunit | 0 | 1 |
|  | Ethanolamine ammonia-lyase, large subunit | 0 | 1 |
| Only in Ellin345 |  |  |  |
|  | Gamma-glutamyl phosphate reductase | 1 | 0 |
|  | N-methylhydantoinase A/acetone carboxylase, beta subunit | 1 | 0 |
|  | N-methylhydantoinase B/acetone carboxylase, alpha subunit | 1 | 0 |
|  | Glutamate 5-kinase | 1 | 0 |
|  | Pyrroline-5-carboxylate reductase | 1 | 0 |
|  | Carbamate kinase | 1 | 0 |
|  | Phosphoserine phosphatase | 2 | 0 |
|  | 3-deoxy-D-arabino-heptulosonate 7-phosphate (DAHP) synthase | 1 | 0 |
|  | Aspartate ammonia-lyase | 1 | 0 |
|  | S-adenosylmethionine decarboxylase | 1 | 0 |
|  | Saccharopine dehydrogenase and related proteins | 1 | 0 |
|  | Predicted Zn peptidase | 2 | 0 |
|  | Tryptophan 2,3-dioxygenase (vermilion) | 1 | 0 |

*Only those categories showing pronounced differences between Ellin6076 and Ellin345 are shown.
