# Supplementary material for: Biological Consequences of Ancient Gene Acquisition and Duplication in the Large Genome of Candidatus Solibacter usitatus Ellin6076
Source: PLoS One. 2011 Sep 15;6(9):e24882. doi: 10.1371/journal.pone.0024882 (PMC3174227; doi:10.1371/journal.pone.0024882)
Supplement: Table S9 — Expansion of COG categories for inorganic transport and metabolism. (DOC) [file pone.0024882.s016.doc]

**Table S9**. Expansion of COG categories for inorganic transport and metabolism.

| Function | genes | # copies in Ellin345 | # copies in Ellin6076 |
| --- | --- | --- | --- |
| Total all functions | all genes in inorganic metabolism COG category | 118 | 179 |
| Specific functions* |  |  |  |
| Enterochelin esterase and related enzymes ( EC:3.1.1.17 ) |  | 2 | 16 |
| Outer membrane receptor proteins, mostly Fe transport |  | 6 | 13 |
|  | TonB-dependent receptor | 3 | 12 |
|  | General function only | 3 | 1 |
|  |  |  |  |
| Outer membrane receptor for ferrienterochelin and colicins |  | 3 | 7 |
|  | TonB-dependent receptor | 2 | 3 |
|  | Cna B-type protein | 1 | 2 |
|  | General function only |  | 2 |
| Mn2+ and Fe2+ transporters of the NRAMP family |  | 3 | 6 |
|  | Mn2+/Fe2+ transporter, NRAMP family | 3 | 1 |
|  | natural resistance-associated macrophage protein | 0 | 5 |
| Only in Ellin6076 |  |  |  |
|  | Arylsulfatase A and related enzymes ( EC:3.1.6.1 ) | 0 | 18 |
|  | Cytochrome c peroxidase ( EC:1.11.1.5 ) | 0 | 6 |
|  | Adenylylsulfate kinase and related kinases | 0 | 2 |
|  | Arsenate reductase and related proteins, glutaredoxin family | 0 | 2 |
|  | Rhodanese-related sulfurtransferase | 0 | 2 |
|  | ABC-type Co2+ transport system, permease component | 0 | 1 |
|  | Di- and tricarboxylate transporters | 0 | 1 |
|  | ABC-type cobalt transport system, permease component CbiQ and related transporters | 0 | 1 |
|  | Putative heme iron utilization protein | 0 | 1 |
|  | Catalase | 0 | 1 |
|  | ABC-type metal ion transport system, periplasmic component/surface adhesin | 0 | 1 |
|  | ABC-type Mn2+/Zn2+ transport systems, permease components | 0 | 1 |
|  | ABC-type cobalt transport system, ATPase component | 0 | 1 |
|  | ABC-type dipeptide/oligopeptide/nickel transport system, ATPase component | 0 | 1 |
|  | Periplasmic molybdate-binding protein/domain | 0 | 1 |
|  | Fe2+ transport system protein A | 0 | 1 |
|  | Cu/Zn superoxide dismutase | 0 | 1 |
|  | Predicted flavoprotein involved in K+ transport | 0 | 1 |
|  | Copper chaperone | 0 | 1 |
|  | Uncharacterized protein affecting Mg2+/Co2+ transport | 0 | 1 |
|  | Fe2+-dicitrate sensor, membrane component | 0 | 1 |
|  | Predicted ferric reductase | 0 | 1 |
|  |  |  |  |
| Only in Ellin345 | ABC-type phosphate transport system, permease component | 1 | 0 |
|  | ABC-type phosphate transport system, permease component | 1 | 0 |
|  | Phosphate uptake regulator | 2 | 0 |
|  | DNA-binding ferritin-like protein (oxidative damage protectant) | 2 | 0 |
|  | ABC-type phosphate transport system, ATPase component | 1 | 0 |
|  | Putative copper export protein | 1 | 0 |
|  | ABC-type Na+ efflux pump, permease component | 1 | 0 |
|  | Protein implicated in iron transport, frataxin homolog | 1 | 0 |
|  | Chromate transport protein ChrA | 1 | 0 |
|  | Membrane transporters of cations and cationic drugs | 1 | 0 |
|  | Dissimilatory sulfite reductase (desulfoviridin), gamma subunit | 1 | 0 |
|  | Na+/H+ antiporter | 1 | 0 |
|  | Nitrous oxidase accessory protein | 1 | 0 |
|  | ABC-type molybdate transport system, ATPase component | 1 | 0 |

*Only those categories showing pronounced differences between Ellin6076 and Ellin345 are shown.
